# Fungal CSL transcription factors

**DOI:** 10.1186/1471-2164-8-233

**Published:** 2007-07-13

**Authors:** Martin Převorovský, František Půta, Petr Folk

**Affiliations:** 1 Department of Physiology and Developmental Biology, Faculty of Science, Charles University in Prague, Viničná 7, CZ 128 44, Praha 2, Czech Republic

## Abstract

**Background:**

The CSL (CBF1/RBP-Jκ/Suppressor of Hairless/LAG-1) transcription factor family members are well-known components of the transmembrane receptor Notch signaling pathway, which plays a critical role in metazoan development. They function as context-dependent activators or repressors of transcription of their responsive genes, the promoters of which harbor the GTG(G/A)GAA consensus elements. Recently, several studies described Notch-independent activities of the CSL proteins.

**Results**

We have identified putative CSL genes in several fungal species, showing that this family is not confined to metazoans. We have analyzed their sequence conservation and identified the presence of well-defined domains typical of genuine CSL proteins. Furthermore, we have shown that the candidate fungal protein sequences contain highly conserved regions known to be required for sequence-specific DNA binding in their metazoan counterparts. The phylogenetic analysis of the newly identified fungal CSL proteins revealed the existence of two distinct classes, both of which are present in all the species studied.

**Conclusion:**

Our findings support the evolutionary origin of the CSL transcription factor family in the last common ancestor of fungi and metazoans. We hypothesize that the ancestral CSL function involved DNA binding and Notch-independent regulation of transcription and that this function may still be shared, to a certain degree, by the present CSL family members from both fungi and metazoans.

## Background

The CSL (CBF1/RBP-Jκ/Suppressor of Hairless/LAG-1) proteins compose a family of transcription factors essential for metazoan development [1,2]. They are present in all metazoan genomes studied and show remarkable sequence conservation across phylogeny. They localize predominantly or exclusively in the cell nucleus where they can either repress or activate transcription depending on the context and the presence of various coregulators. CSL proteins recognize a very tightly defined consensus sequence GTG(G/A)GAA in target promoters. Their best characterized function relates to the signaling pathway of the transmembrane receptor Notch where they mediate the effector nuclear step – activation of Notch-responsive genes. The Notch pathway regulates metazoan embryonic development, cell fate decisions and tissue boundaries specifications [2,3]. Its deregulation is implicated in several diseases including cancer [4] and, in addition, several viruses encode factors that misuse this pathway via interaction with CSL proteins [5].

CSL proteins are essential for the development of the organism as a whole, however, they are dispensable at the cellular level, because CSL knock-out cell lines can be established and do not show any obvious abnormalities. The mutant phenotypes of Notch and CSL genes do not fully overlap, as CSL mutants show more severe developmental perturbations [2,6]. Recently, several studies reported Notch-independent activities of CSL proteins indicative of their involvement in yet other signaling pathways [7-10]. In addition to the Notch pathway-dependent CSL proteins of the RBP-Jκ type, at least in some metazoan species, CSL transcription factors called RBP-L can be found, which are only beginning to be characterized. They are highly similar to the RBP-Jκ group but seem to act exclusively in a Notch-independent manner. Unlike the ubiquitous RBP-Jκ type proteins the expression of RBP-L is confined to only a few tissue types [11,12].

In contrast to the generally accepted view, the presence of CSL proteins seems not to be confined to metazoan organisms and the Notch pathway. They are indeed absent from plants but there were indications of CSL proteins in one fungal species – the fission yeast *Schizosaccharomyces pombe *[13]. We have attempted to confirm the identity of CSL proteins in *S. pombe *and to further explore the distribution of this transcription factor family in fungi. We have documented the existence of fungal CSL proteins, which indicates that this family originated much earlier in evolution than previously appreciated. We hope that these findings will help to elucidate the CSL family ancestral function in cells and to better understand their complex engagements in metazoans.

## Results

### Identification of CSL genes in fungi

CSL transcription factors are generally considered a key part of the Notch signaling pathway and as such a hallmark of metazoan organisms [2]. However, it was noted earlier in the literature that distant CSL homologs may also be found in the genome of the fission yeast *Schizosaccharomyces pombe*, an organism that lacks the Notch pathway [13]. This raises interesting questions regarding the evolutionary origin as well as the ancestral function of the CSL family. We have therefore conducted exhaustive BLAST searches of publicly available sequence data (see Methods) to asses the presence and conservation of CSL family members in fungi. The results of these searches are summarized in Table 1 (the fungal taxonomical nomenclature used in this article was taken from [14]). Nineteen putative CSL genes were found in seven organisms, with *S. pombe *and *S. japonicus *belonging to the Taphrinomycotina basal subphylum of ascomycetes, *Rhizopus oryzae *representing the zygomycetes and *Coprinus cinereus*, *Cryptococcus neoformans*, *Phanerochaete chrysosporium *and *Ustilago maydis *belonging to the basidiomycetes. Protein products of these genes contain motifs typical of the CSL family (see below). It is likely that more CSL genes will be found in these taxonomical groups as more genome sequences become available. In contrast, no CSL homologs could be found in either Saccharomycotina (including the budding yeast *Saccharomyces cerevisiae*) or Pezizomycotina, the later branching subphyla of ascomycetes.

**Table 1:** Fungal CSL proteins

**Organism**	**Protein**	**Accession number/Locus**^a^	**Length (aa)**	**Status**^b^	**Nuclear**^c^	**Source**
**Ascomycota: Taphrinomycotina**						

***Schizosaccharomyces pombe***	SPCC1223.13	CAA20882	963	Exp^d^	+++	[40]
	SPCC736.08	NP_587779	613	Exp^d^	-	[40]
***Schizosaccharomyces japonicus***	SjCSL2	Supercontig 4 (bases 1104530–1107169)	879	Hyp	+++	[31]^e^
	SjCSL1	Supercontig 5 (bases 726033–727712)	559	Hyp	+++	[31]^e^

**Zygomycota**						

***Rhizopus oryzae***	RO3G_06481	RO3G_06481.1	694	Hyp	+++	[51]
	RO3G_07636	RO3G_07636.1	662	Hyp	++	[51]^e^
	RO3G_11583	RO3G_11583.1	764	Hyp	+++	[51]^e^
	RO3G_14587	RO3G_14587.1	886	Hyp	+++	[51]^e^
	RO3G_06953	RO3G_06953.1	449	Hyp	+++	[51]
	RO3G_08863	RO3G_08863.1	482	Hyp	+++	[51]
	RO3G_13784	RO3G_13784.1	478	Hyp	+++	[51]

**Basidiomycota**						

***Coprinus cinereus***	CC1G_03194	EAU91026	960	Hyp	+++	[40]
	CC1G_01706	CC1G_01706.1	803	Hyp	+	[52]^e^
***Cryptococcus neoformans***	CNBD3370	EAL21283	1015	Hyp	+++	[40]
	CNA01890	AAW40742	776	Exp	+++	[40]
***Phanerochaete chrysosporium***	PcCSL2	Scaffold 6 Contig 19 (bases 50978–54385)	1012	Hyp	++	[53]^e^
	Pc6518	protein id "6518"	960	Hyp	++	[53]
***Ustilago maydis***	UM06280	EAK82808	1482	Hyp	+++	[40]
	UM05862	EAK86807	1094	Hyp	+++	[40]

Notes: ^a^refers to the respective database stated in the 'Source' column; ^b^Exp – expressed protein, Hyp – hypothetical protein; ^c^nuclear localization prediction score (see Methods); ^d^MP et al., manuscript in preparation; ^e^see Additional files 1 and 2 for new sequence predictions and corrections made to the original database sequences.

Most of the candidates are hypothetical proteins with little or no annotation in the databases. Therefore, we have first verified the quality of each ORF prediction (see Methods). The confidence of exon-intron structure predictions in these less studied organisms is rather limited. Another obstacle is posed by the degree of divergence among the sequences together with the presence of multiple species- and protein-specific insertions. Nevertheless, we were able to construct three completely new gene predictions (designated SjCSL1 and SjCSL2 in *S. japonicus*, and PcCSL2 in *P. chrysosporium*) as well as to identify mispredictions and/or possible sequencing errors in other four genes (see Additional files 1 and 2 for a more detailed description). Our corrections comprised of intron inclusion/exclusion, different splice-site selection and exon addition. Some of the intron positions displayed inter-species conservation which supported our predictions (data not shown). We have also identified a less usual intron with a GC-AG boundary in the *R. oryzae *RO3G_07636.1 gene. Such introns were found in other fungi as well [15] and are generally a problem for gene prediction algorithms.

Typically, there are two CSL paralogs per genome, differing considerably in length and each belonging to a different class (see below). A notable exception is the genome of *R. oryzae *which harbors seven CSL genes, three of them being class F1 and four of them belonging to class F2. Most candidate CSL proteins are predicted to be nuclear which supports their putative functioning as transcription factors (see bellow). SPCC736.08 of *S. pombe *is the only protein predicted to have exclusively non-nuclear subcellular localization but it was shown experimentally to be nuclear [16].

### Sequence conservation of fungal CSL proteins

According to the *C. elegans *LAG-1 protein crystal structure, the CSL fold is related to Rel-domain proteins, but is uniquely composed of three distinct domains [17]. The amino-terminal RHR-N (Rel-homology region) and central BTD (beta-trefoil domain) domains are involved in DNA-binding. BTD serves also as an interaction platform for Notch/SMRT coregulators. The carboxy-terminal RHR-C domain displays lower conservation in metazoans and its function is not yet clear; one possibility is its participation in Notch-independent regulation of transcription [18].

**Figure 1:**
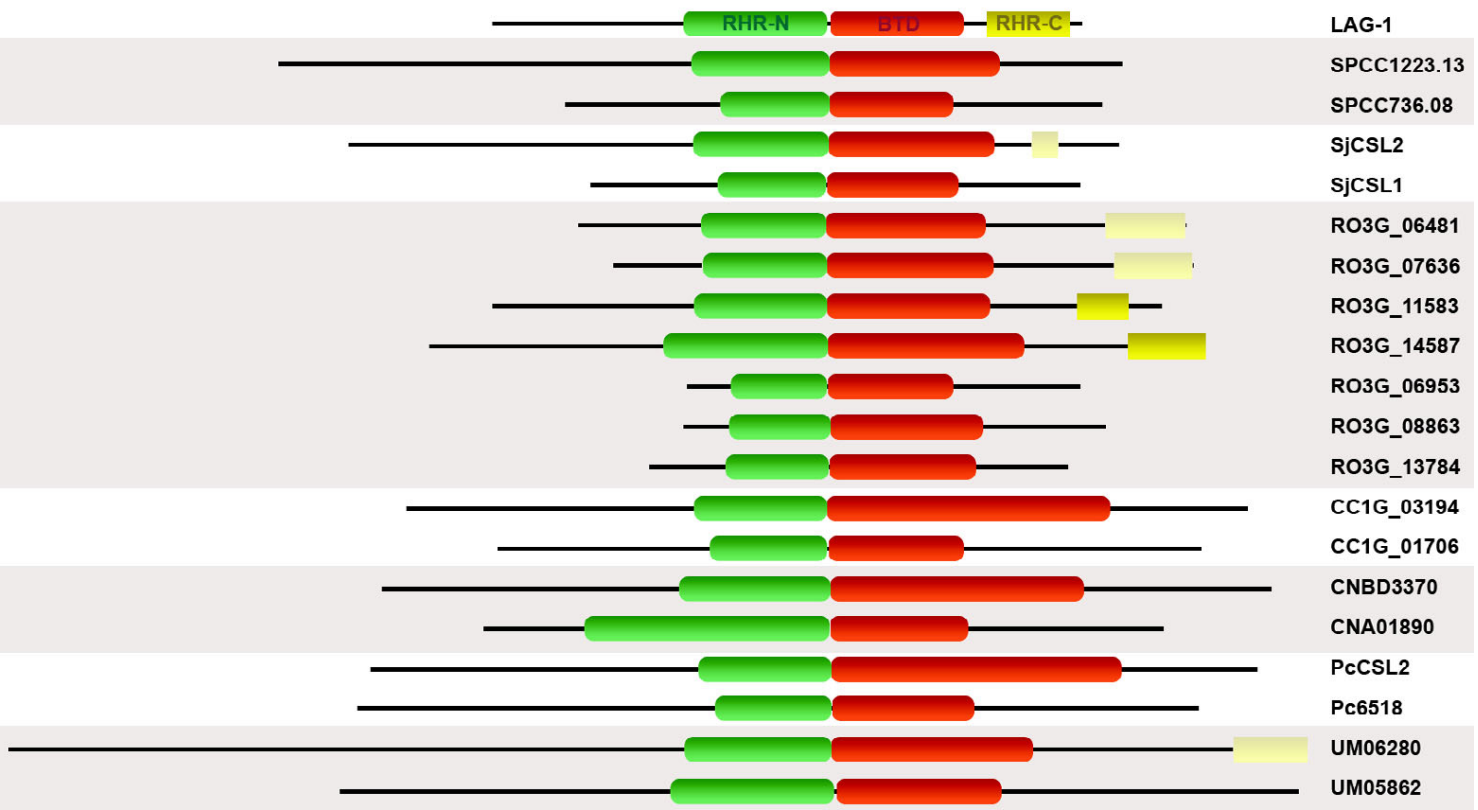
**Fungal CSL proteins domain organization**. Black lines represent the respective CSL protein sequences (see Table 1 for details). The structure of *C. elegans *LAG-1 is shown at the top for comparison [17]. Recognized Pfam domains are indicated: RHR-N in green, BTD in red and RHR-C in yellow (light yellow for low significance). The proteins are drawn to scale.

We have used the Pfam protein domains database [19] to search for CSL-specific domains in all our candidate sequences and to identify any other known domains present. The results are schematized in Fig. 1. The RHR-N [Pfam:PF09271] and BTD [Pfam:PF09270] domains were identified in all fungal sequences with high significance, supporting the identity of our candidates as CSL family members. However, the RHR-C [Pfam:PF01833] domain could only be identified in RO3G_11583 and RO3G_14587 from *R. oryzae*. A rather divergent RHR-C domain was also found in *S. japonicus *SjCSL2 and two more *R. oryzae *proteins, RO3G_06481 and RO3G_07636. The lower degree of sequence conservation of RHR-C noted in metazoans is thus even more pronounced in fungi. No other conserved domains could be found, despite the fact that the putative fungal CSL proteins are typically significantly larger than their metazoan counterparts. The overall domain organization of the fungal proteins is the same as in metazoans. The increased size of the fungal candidates was found to be caused by two factors. First, in some proteins, there are pronounced extensions of the amino-terminal part preceding the RHR-N domain. This region is about 200 amino acids long in *C. elegans *and gets much shorter in metazoan evolution. Its crystal structure is not known. Second, there are multiple amino acid insertions of varying length throughout the candidate sequences (see below).

**Figure 2:**
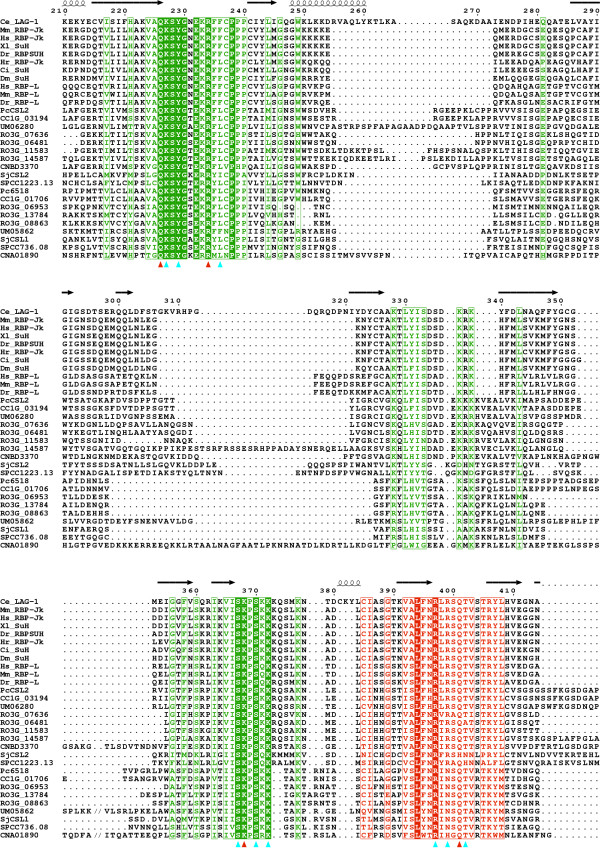
**Evolutionary conservation of the DNA-binding regions**. The alignment of fungal and selected metazoan CSL protein sequences (see Table 1 and Additional file 3 for details) shows high degree of conservation in regions responsible for DNA binding. Absolutely conserved residues are inverse-printed, positions with high residue similarity are boxed. Domain boundaries are indicated by color: green for RHR-N, red for BTD and blue for the βC4 linker connecting all three CSL domains. Red and cyan triangles below the alignment denote residues required for sequence specific and backbone DNA binding, respectively. The position numbering and secondary structures indicated above the sequences correspond to *C. elegans *LAG-1 [17]. The picture shows only a selected region of the whole alignment and, in order to save space, some parts of the long inserts are not shown (indicated by '//'). The picture was created using ESPript [50].

**Figure 3:**
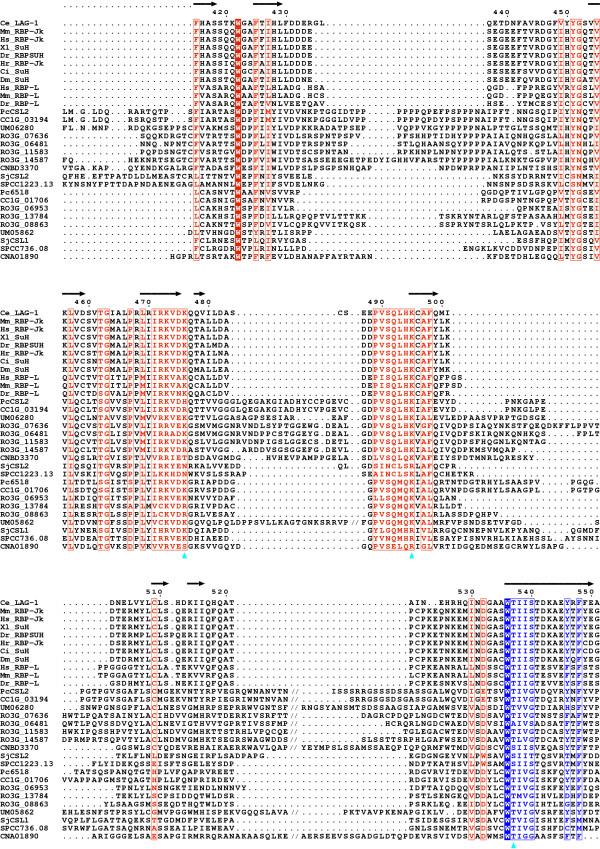
**Evolutionary conservation of the DNA-binding regions – continued**. The continuation of the alignment shown in Fig. 2.

To gain better insight into the specifics of the fungal CSL proteins, we have produced a multiple sequence alignment of all newly identified fungal sequences and selected metazoan family members (see Methods and Additional file 3). There are two sub-types of metazoan CSL proteins; one is represented by the Notch-pathway protein RBP-Jκ (CBF1, SuH, RBPSUH) and the other by the much less known transcription factor RBP-L, the function of which seems to be Notch-independent [11,12]. Both subtypes' representatives were included in the alignment. The most prominent feature of the resulting alignment is the presence of several highly conserved blocks of amino acids separated by species- and protein-specific insertions. These insertions are of considerable length in some cases and are more pronounced in the class F2 proteins. They are rich in amino acids proline, glycine, serine/threonine and lysine/arginine. Overall sequence conservation is highest in the RHR-N and BTD domains, including the immediately following long β-strand (βC4) that was shown to bridge all three CSL domains in the *C. elegans *LAG-1 [17]. The conservancy of the βC4 linker suggests that the CSL-specific arrangement between RHR-N and BTD is also likely preserved in fungi. The C-termini typically contain only 1–2 well-alignable stretches that can be identified as fragments of the RHR-C domain. The amino-terminal extensions preceding the RHR-N domain show little if any sequence conservation. As mentioned above, there are several regions located mostly in the RHR-N and BTD domains, that show very high or even absolute sequence conservation (see Fig. 2 and 3). It is notable that, according to the crystallography data, all these conserved blocks are involved in binding of the strictly defined CSL consensus site on DNA [17]. With the sole exception of the *S. japonicus *SjCSL2 protein (Q567H substitution corresponding to Q401 in *C. elegans *LAG-1, see Fig. 2), all residues required for sequence specific binding of the GTG(G/A)GAA response element are absolutely conserved in all fungal proteins, which strongly supports their inclusion in the CSL family. The interactions of CSL proteins with their coactivators Notch/EBNA2 and corepressors SMRT/NCoR and CIR have been mapped to and around a hydrophobic pocket on the surface of BTD [17,20-22]. Not surprisingly, the residues mediating these interactions are generally not conserved in fungi, although some of them are found in class F2 fungal CSL proteins. However, the potential to form a hydrophobic pocket in BTD seems to be preserved (data not shown).

### Phylogenetic analysis of the CSL protein family

**Figure 4:**
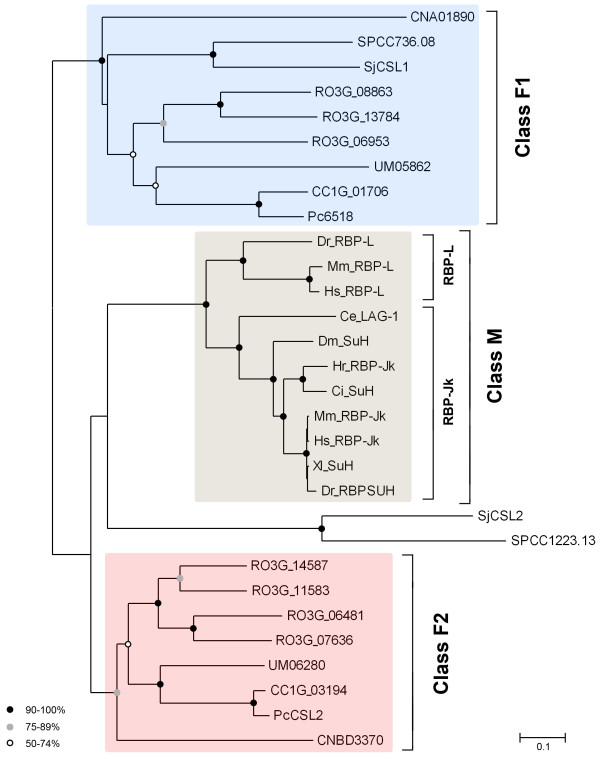
**Phylogenetic analysis of the CSL protein family**. An unrooted neighbor-joining phylogenetic tree of the region corresponding to RHR-N and BTD domains (see Methods). For protein descriptions see Table 1 and Additional file 3. For class F2 only the unambiguous core, not including the *S. pombe *SPCC1223.13 and *S. japonicus *SjCSL2, is indicated by shading. Symbols at nodes indicate percentual bootstrap values, no symbol means less than 50% node stability. The scale bar indicates the number of amino acid substitutions per site.

As noted earlier, there are usually two fungal CSL paralogs per genome. We wanted to see whether these paralogs cluster to some well-defined groups and what their relationship to the metazoan CSL family members is. For this purpose, we have constructed an unrooted phylogenetic tree for the regions that could be aligned with confidence, that is, the RHR-N and BTD domains (see Methods and Fig. 4). As expected, the fungal CSL proteins form two distinct classes, designated class F1 and F2, with each class being represented in all fungal taxons included in the analysis. It should be noted at this point that the positions of *S. pombe *SPCC1223.13 and *S. japonicus *SjCSL2 proteins are slightly ambiguous, branching off either immediately before or after the class F2 core (data not shown). The intra-class branch topology roughly follows the taxonomical relations [23] with the notable exception of the divergent *C. neoformans *CNA01890 and CNBD3370 proteins. It can be inferred from the branch lengths that the rate of divergence among the fungal protein sequences is much higher than in metazoa. Metazoan CSL proteins (designated class M) form a very coherent group that can be divided to RBP-Jκ and RBP-L subgroups. The RBP-Jκ subgroup displays an especially low extent of divergence, which may be due to their involvement in the developmentally critical Notch pathway. Of the two fungal CSL classes the class F2 proteins show higher similarity to the metazoan class M.

## Discussion

### The CSL family origin and distribution

To the best of our knowledge, there were only two brief notions of CSL proteins existence outside metazoans up to now. One paper showed Southern blot cross-hybridization of murine RBP-Jκ cDNA probe with *S. pombe *DNA [24]. The significance of these results is, however, questionable, as the hybridizing chromosomal DNA fragments had lengths differing from that expected for either of *S. pombe *CSL genes, SPCC736.08 and SPCC1223.13. Potential CSL homologs in *S. pombe *were also mentioned in the review of Lai [13], although no supporting evidence was presented.

We have rigorously searched for CSL proteins in eukaryotic genomes from all kingdoms of life to map their distribution. Apart from the known metazoan proteins, we have found no homologs in either plants or protozoa (data not shown), however, we have succeeded in finding CSL family members in several fungal species of the ascomycetes (the basal subphylum Taphrinomycotina), zygomycetes and basidiomycetes groups. These organisms range in complexity from the simple unicellular fission yeast to the macroscopic multicellular and highly differentiated *C. cinereus*. It is of notion that the presence of CSL homologs in fungi is not universal as there are no representatives found in either of the later branching ascomycetal groups, Saccharomycotina, including the important model organism *S. cerevisiae*, and Pezizomycotina. Our data support the idea that the ancestral CSL gene originated in the last common ancestor of animals and fungi, thus much earlier than previously assumed. This is in accord with the absence of CSL family in such large groups as plants and mycetozoa, that branched off earlier in evolution [25,26]. We hypothesize that the first CSL gene might have been created from a Rel-type transcription factor gene by the insertion of a beta-trefoil domain-encoding DNA sequence in between the amino- and carboxy-terminal Rel domains. Subsequently, a duplication event took place in the fungal lineage creating the two CSL classes we see today, class F2 being more alike the metazoan CSL proteins and class F1 being more fungi-specific (see Fig. 4). We consider such explanation more likely than the alternative, where the ancestral CSL gene would both originate and undergo duplication in the common ancestor of metazoans and fungi and one copy would be soon lost again in the metazoan lineage.

Nevertheless, there have been independent losses of CSL genes in the fungal branch. First, we failed to find any CSL homologs in *Encephalitozoon cuniculi *(data not shown), a parasitic microsporidian and a representative of a group that is sister to fungi [25]. This fact is probably due to the parasitic lifestyle of these organisms, which often leads to pronounced gene eliminations [27]. Second, we have found no evidence of CSL genes in chytridiomycetes (data not shown), a likely polyphyletic group also basal to the fungal lineage [14]. Finally, the CSL family is apparently missing in the later branching ascomycetal fungi of the Saccharomycotina and Pezizomycotina groups [23], suggestive of another gene loss(es). The losses may have occurred during the transitions between saprophytic and parasitic nutritional modes [14], indicating that the CSL genes code for functions in fungi that are not universally required in their life cycles. On the other hand, there have been clade specific CSL genes multiplications in fungi illustrated by the three class F1 and four class F2 CSL genes of *Rhizopus oryzae*. Evolutionary pressure could have favored proliferation and diversification of the CSL family in this branch of zygomycetes, similarly to the expansions that were documented for other gene families and phyla, such as, e.g., nuclear hormone receptors and nematodes, or calmodulin-type proteins and dictyostelids, respectively [28,29]. A history of gene losses and duplications in the fungal lineage has also been described for proteins involved in various RNA silencing phenomena [30]. The metazoan CSL genes (class M) obviously underwent duplication too. It likely occurred in the common ancestor of all vertebrates and gave rise to the RBP-L type of proteins, in addition to the RBP-Jκ type universally present in both vertebrate and invertebrate animals. It should be noted in this regard, that the RBP-L type gene is present in zebrafish, but so far no homologs have been reported in the genetically rather complicated clawed frog *Xenopus laevis*. We have also failed to identify an RBP-L homolog in the more tractable species *X. tropicalis*, thus amphibians likely have developed ways to regulate all their CSL-responsive genes using the RBP-Jκ homolog only. In summary, we have found representatives of the important transcription factor family CSL, up to now generally considered metazoan-only, in several groups of fungi and showed that they are an ancient gene family that originated much earlier than their current metazoan affiliates like Notch or Mastermind [13].

### The conservation of fungal CSL proteins

The degree of conservation of CSL proteins across phylogeny is remarkable, given the evolutionary distances, and points to an important role they likely play in cells [25]. The sequence similarity among metazoan CSL proteins is extremely high and does not allow for finding functionally important regions directly from sequence comparison. On the other hand, the distant CSL homologs from fungi may provide this information more readily. Indeed, we have found that the most prominent conservation can be found in the regions involved in DNA binding with the critical residues and several motifs being invariant in all proteins analyzed (see Fig. 2 and 3). As expected, when compared to metazoans, the rate of divergence has been much faster in fungi, especially in those having small genomes, i.e. *C. neoformans*, *S. pombe *and *S. japonicus *[31-33]. In fact, the *C. neoformans *CSL proteins are the most divergent ones among fungi and their position in our phylogenetic tree (Fig. 4) differs from that expected by looking at the fungal tree of life [23]. Such discrepancy has also been reported for other *C. neoformans *proteins [30] and it has been demonstrated for *S. pombe *that various types of proteins might produce inconsistent signals when used for phylogenetic analyses [34].

There are numerous insertions separating the above-mentioned conserved sequence stretches, but these insertions are often rich in amino acids that are likely to appear in loops and solvent-exposed regions [35]. In addition, such insertions are present, to a lesser degree, also in the *C. elegans *LAG-1, the most evolutionarily primitive CSL protein studied so far [17]. It may be argued that the fungal insertions could be an artifact produced by ORF misprediction. We cannot rule out this possibility completely as the tools for identifying exon-intron boundaries optimized for diverse fungal species are limited or lacking. However, many of these insertions are conserved among the classes of CSL proteins and their positions mostly correspond to the LAG-1 loops and regions exposed on the surface of the protein [17]. Thus the general CSL fold may be well preserved in fungi.

Furthermore, the splicing pattern of some fungal CSL genes is partially conserved among species (data not shown) and the ORF predictions used in this study are in good agreement with the multiple sequence alignment of the proteins they encode. Nevertheless, the prediction reliability of the non-conserved amino-terminal extensions found in some fungal CSL proteins remains questionable. The sequence similarity in the parts of the fungal proteins corresponding to known coregulator interaction sites in metazoans seems not to be significantly preserved. This is of no great surprise as these coregulators are frequently involved in the Notch signaling pathway, which is lacking in fungi, or are encoded by mammalian viruses [5,13]. Also, the less-conserved metazoan RHR-C domain of yet unknown function is very loosely defined in fungi, as it was identified with confidence only in several class F2 members. Taken together, our data suggest that the fungal CSL proteins may adopt the CSL fold and we further show that these proteins posses notably conserved regions of functional significance related mostly to their ability to bind DNA in a sequence-specific manner.

### The ancestral role of the CSL transcription factor family

Our current knowledge of the CSL family derives exclusively from metazoan model organisms and is based mostly on studies concerning development and the Notch pathway [2,9,13]. It is now clear that this is not the whole picture as we have presented evidence of CSL proteins in several organisms that are evolutionarily distant to animals and lack the critical Notch pathway components. Moreover, recent reports on metazoan model organisms indicate, that there are yet unrecognized CSL activities in animals as well [7,8,10,11]. It is tempting to speculate that the CSL ancestral function is preserved in the fungal proteins of today and maybe even in metazoans, where it might be responsible for some of the Notch-independent activities observed. If this is the case we would have excellent models, e.g., the genetically tractable fission yeast *S. pombe*, to study it.

We hypothesize that the ancestral function is likely the regulation of gene expression, where other signals than Notch receptor activation are interpreted. Our first clue comes from the analysis of fungal CSL sequence conservation, which clearly indicates their potential to bind DNA. This includes not only DNA binding in general, but goes further to the ability to recognize the strict CSL consensus. The second clue derives from the lack of conservation of CSL interacting partners from metazoans. As stated above, the Notch receptor, its ligands and coactivators are not present in fungi. Finally, the metazoan CSL proteins are essential for embryonic development but dispensable in cultured cells [6]. Similarly, the deletion of either or both *S. pombe *CSL genes is viable (MP et al., manuscript in preparation; and [36,37]). This suggests, together with the secondary loss of CSL genes in some fungi (see above), that the proposed ancestral function in gene regulation is not essential.

We also have to account for the existence of two CSL classes in fungi. There is analogy to the metazoan class M sub-groups, the RBP-Jκ and RBP-L CSL types. Both are involved in transcription regulation, but differ in their interacting partners, their responsiveness to various signals, their expression profiles and their *in vivo *DNA-binding preferences [11,12]. The similar may be true for class F1 and class F2 fungal CSL proteins. They may all participate in transcription regulation, but have either distinct or only partially overlapping target gene sets. Alternatively, they may differentially regulate the same genes, with the outcome depending on, e.g., environmental conditions. It was indeed found by whole-genome microarray experiments, that the *S. pombe *CSL genes display differential expression during sexual differentiation and under various stress conditions [38,39]. In conclusion, the CSL gene family encodes proteins that are likely universally involved in the regulation of transcription both in animals and fungi.

## Conclusion

We have shown the existence of CSL transcription factor family, known from studies of the metazoan Notch signaling pathway, in several fungal species. We have described conserved features of the fungal proteins supporting their identity as true CSL family members. These findings put the CSL family origin further back in evolution, deeper than currently understood. We have mapped the history of CSL gene duplication and gene loss events in the fungal lineage, showing the existence of two well-defined CSL classes, class F1 and class F2, respectively, with the second class being more similar to the metazoan class M proteins. We hypothesize that the ancestral CSL function involved DNA binding and Notch-independent regulation of transcription and that this function may still be shared, to a certain degree, by the present CSL family members from both fungi and metazoans. If true, that would allow for exploiting the simple fungal models to analyze this function. We are currently studying the CSL proteins role in *S. pombe *and experiments are underway to identify the sets of genes and processes they regulate.

## Methods

### Database searches for CSL genes

We have searched multiple publicly available fungal genome and protein databases (including NCBI [40] and UniProt [41]) using the appropriate BLAST algorithm with default settings and with the mouse CBF1 protein [GenBank:NP_033061] as a query. Candidate hits containing at least one of the conserved CSL motifs (see Results) were considered and used for further analyses. The BLAST searches were then repeated with all the newly identified CSL sequences as queries until no more new hits were found. In cases where two or more nearly-identical candidate sequences, coming from independent sources and obviously representing a single gene, were found, the sequence showing the highest degree of similarity to the fungal CSL consensus was chosen. The final searches were performed between November 24, 2006 and November 30, 2006.

### Gene models prediction and verification

All candidate fungal CSL proteins were checked for the quality of their ORF prediction. We compared each database gene model with GenScan [42] and/or WebGene [43] predictions. The models were also compared to a multiple sequence alignment of other CSL proteins. In some cases, the splicing pattern was corrected manually using the Gene Runner 3.05 software (Hastings Software, Inc.) in order to restore a highly conserved region (see Results and Additional files 1 and 2).

### Conserved domain search and protein localization prediction

Known domains present in the fungal CSL proteins were searched for by the Search Pfam server [19]. Subcellular localization of each CSL protein was predicted by three independent algorithms, namely SubLoc v1.0 [44], CELLO v.2.5 [45] and PSORT II [46]. Each sequence received score ranging from '-' to '+++' depending on the number of times the protein was predicted to be nuclear (see Table 1).

### Sequence alignments and phylogenetic analyses

Alignments used during the sequence retrieval part of the study were performed using ClustalW [47]. The final alignment of all identified fungal and selected metazoan CSL proteins was based on a ClustalX output (Blosum matrix series) [48], which was then manually edited in BioEdit 7.0.5.3 to correct some obvious alignment errors and to account for the information from the *C. elegans *CSL protein crystal structure [17]. See Additional file 3 for the final alignment and the list of metazoan sequences used.

For tree construction all positions containing gaps were removed from the final sequence alignment. An unrooted phylogenetic tree was then generated for the region corresponding to RHR-N and BTD domains (from helix α2 just before the βC4 linker, residues 210–535 in the *C. elegans *LAG-1 reference protein, see [17]) using the neighbor-joining method in the MEGA 3.1 software package [49] with 2000 bootstrap replicates.

## Authors' contributions

MP designed the study, carried out database searches and phylogenetic analyses and drafted the manuscript. FP participated in the study design and final manuscript preparation. PF participated in the final manuscript preparation. All authors read and approved the final manuscript.

## Supplementary Material

Additional file 1 New and corrected fungal CSL gene prediction models Click here for file

Additional file 2 New and corrected fungal CSL protein prediction models Click here for file

Additional file 3 CSL proteins sequence alignment used for the phylogenetic analyses Click here for file

## References

[B1] Pursglove SE, Mackay JP (2005). CSL: a notch above the rest. Int J Biochem Cell Biol.

[B2] Artavanis-Tsakonas S, Rand MD, Lake RJ (1999). Notch signaling: cell fate control and signal integration in development. Science.

[B3] Weinmaster G, Kintner C (2003). Modulation of Notch signaling during somitogenesis. Annu Rev Cell Dev Biol.

[B4] Weng AP, Aster JC (2004). Multiple niches for Notch in cancer: context is everything. Curr Opin Genet Dev.

[B5] Hayward SD (2004). Viral interactions with the Notch pathway. Semin Cancer Biol.

[B6] Oka C, Nakano T, Wakeham A, de la Pompa JL, Mori C, Sakai T, Okazaki S, Kawaichi M, Shiota K, Mak TW, Honjo T (1995). Disruption of the mouse RBP-J kappa gene results in early embryonic death. Development.

[B7] Koelzer S, Klein T (2003). A Notch-independent function of Suppressor of Hairless during the development of the bristle sensory organ precursor cell of Drosophila. Development.

[B8] Kaspar M, Klein T (2006). Functional analysis of murine CBF1 during Drosophila development. Dev Dyn.

[B9] Bray S, Furriols M (2001). Notch pathway: making sense of suppressor of hairless. Curr Biol.

[B10] Barolo S, Walker RG, Polyanovsky AD, Freschi G, Keil T, Posakony JW (2000). A notch-independent activity of suppressor of hairless is required for normal mechanoreceptor physiology. Cell.

[B11] Beres TM, Masui T, Swift GH, Shi L, Henke RM, MacDonald RJ (2006). PTF1 is an organ-specific and Notch-independent basic helix-loop-helix complex containing the mammalian Suppressor of Hairless (RBP-J) or its paralogue, RBP-L. Mol Cell Biol.

[B12] Minoguchi S, Taniguchi Y, Kato H, Okazaki T, Strobl LJ, Zimber-Strobl U, Bornkamm GW, Honjo T (1997). RBP-L, a transcription factor related to RBP-Jkappa. Mol Cell Biol.

[B13] Lai EC (2002). Keeping a good pathway down: transcriptional repression of Notch pathway target genes by CSL proteins. EMBO Rep.

[B14] James TY, Kauff F, Schoch CL, Matheny PB, Hofstetter V, Cox CJ, Celio G, Gueidan C, Fraker E, Miadlikowska J, Lumbsch HT, Rauhut A, Reeb V, Arnold AE, Amtoft A, Stajich JE, Hosaka K, Sung GH, Johnson D, O'Rourke B, Crockett M, Binder M, Curtis JM, Slot JC, Wang Z, Wilson AW, Schussler A, Longcore JE, O'Donnell K, Mozley-Standridge S, Porter D, Letcher PM, Powell MJ, Taylor JW, White MM, Griffith GW, Davies DR, Humber RA, Morton JB, Sugiyama J, Rossman AY, Rogers JD, Pfister DH, Hewitt D, Hansen K, Hambleton S, Shoemaker RA, Kohlmeyer J, Volkmann-Kohlmeyer B, Spotts RA, Serdani M, Crous PW, Hughes KW, Matsuura K, Langer E, Langer G, Untereiner WA, Lucking R, Budel B, Geiser DM, Aptroot A, Diederich P, Schmitt I, Schultz M, Yahr R, Hibbett DS, Lutzoni F, McLaughlin DJ, Spatafora JW, Vilgalys R (2006). Reconstructing the early evolution of Fungi using a six-gene phylogeny. Nature.

[B15] Rep M, Duyvesteijn RG, Gale L, Usgaard T, Cornelissen BJ, Ma LJ, Ward TJ (2006). The presence of GC-AG introns in Neurospora crassa and other euascomycetes determined from analyses of complete genomes: implications for automated gene prediction. Genomics.

[B16] Matsuyama A, Arai R, Yashiroda Y, Shirai A, Kamata A, Sekido S, Kobayashi Y, Hashimoto A, Hamamoto M, Hiraoka Y, Horinouchi S, Yoshida M (2006). ORFeome cloning and global analysis of protein localization in the fission yeast Schizosaccharomyces pombe. Nat Biotechnol.

[B17] Kovall RA, Hendrickson WA (2004). Crystal structure of the nuclear effector of Notch signaling, CSL, bound to DNA. EMBO J.

[B18] Tang Z, Kadesch T (2001). Identification of a novel activation domain in the Notch-responsive transcription factor CSL. Nucleic Acids Res.

[B19] Finn RD, Mistry J, Schuster-Bockler B, Griffiths-Jones S, Hollich V, Lassmann T, Moxon S, Marshall M, Khanna A, Durbin R, Eddy SR, Sonnhammer EL, Bateman A (2006). Pfam: clans, web tools and services. Nucleic Acids Res.

[B20] Sakai T, Taniguchi Y, Tamura K, Minoguchi S, Fukuhara T, Strobl LJ, Zimber-Strobl U, Bornkamm GW, Honjo T (1998). Functional replacement of the intracellular region of the Notch1 receptor by Epstein-Barr virus nuclear antigen 2. J Virol.

[B21] Fuchs KP, Bommer G, Dumont E, Christoph B, Vidal M, Kremmer E, Kempkes B (2001). Mutational analysis of the J recombination signal sequence binding protein (RBP-J)/Epstein-Barr virus nuclear antigen 2 (EBNA2) and RBP-J/Notch interaction. Eur J Biochem.

[B22] Hsieh JJ, Zhou S, Chen L, Young DB, Hayward SD (1999). CIR, a corepressor linking the DNA binding factor CBF1 to the histone deacetylase complex. Proc Natl Acad Sci U S A.

[B23] Kuramae EE, Robert V, Snel B, Weiss M, Boekhout T (2006). Phylogenomics reveal a robust fungal tree of life. FEMS Yeast Res.

[B24] Furukawa T, Kawaichi M, Matsunami N, Ryo H, Nishida Y, Honjo T (1991). The Drosophila RBP-J kappa gene encodes the binding protein for the immunoglobulin J kappa recombination signal sequence. J Biol Chem.

[B25] Hedges SB (2002). The origin and evolution of model organisms. Nat Rev Genet.

[B26] Ciccarelli FD, Doerks T, von MC, Creevey CJ, Snel B, Bork P (2006). Toward automatic reconstruction of a highly resolved tree of life. Science.

[B27] Katinka MD, Duprat S, Cornillot E, Metenier G, Thomarat F, Prensier G, Barbe V, Peyretaillade E, Brottier P, Wincker P, Delbac F, El AH, Peyret P, Saurin W, Gouy M, Weissenbach J, Vivares CP (2001). Genome sequence and gene compaction of the eukaryote parasite Encephalitozoon cuniculi. Nature.

[B28] Sluder AE, Maina CV (2001). Nuclear receptors in nematodes: themes and variations. Trends Genet.

[B29] Rosel D, Puta F, Blahuskova A, Smykal P, Folk P (2000). Molecular characterization of a calmodulin-like dictyostelium protein CalB. FEBS Lett.

[B30] Nakayashiki H, Kadotani N, Mayama S (2006). Evolution and diversification of RNA silencing proteins in fungi. J Mol Evol.

[B31] Schizosaccharomyces japonicus Sequencing Project. Broad Institute of MIT and Harvard. http://www.broad.mit.edu/annotation/genome/schizosaccharomyces_japonicus.

[B32] Wood V, Gwilliam R, Rajandream MA, Lyne M, Lyne R, Stewart A, Sgouros J, Peat N, Hayles J, Baker S, Basham D, Bowman S, Brooks K, Brown D, Brown S, Chillingworth T, Churcher C, Collins M, Connor R, Cronin A, Davis P, Feltwell T, Fraser A, Gentles S, Goble A, Hamlin N, Harris D, Hidalgo J, Hodgson G, Holroyd S, Hornsby T, Howarth S, Huckle EJ, Hunt S, Jagels K, James K, Jones L, Jones M, Leather S, McDonald S, McLean J, Mooney P, Moule S, Mungall K, Murphy L, Niblett D, Odell C, Oliver K, O'Neil S, Pearson D, Quail MA, Rabbinowitsch E, Rutherford K, Rutter S, Saunders D, Seeger K, Sharp S, Skelton J, Simmonds M, Squares R, Squares S, Stevens K, Taylor K, Taylor RG, Tivey A, Walsh S, Warren T, Whitehead S, Woodward J, Volckaert G, Aert R, Robben J, Grymonprez B, Weltjens I, Vanstreels E, Rieger M, Schafer M, Muller-Auer S, Gabel C, Fuchs M, Dusterhoft A, Fritzc C, Holzer E, Moestl D, Hilbert H, Borzym K, Langer I, Beck A, Lehrach H, Reinhardt R, Pohl TM, Eger P, Zimmermann W, Wedler H, Wambutt R, Purnelle B, Goffeau A, Cadieu E, Dreano S, Gloux S, Lelaure V, Mottier S, Galibert F, Aves SJ, Xiang Z, Hunt C, Moore K, Hurst SM, Lucas M, Rochet M, Gaillardin C, Tallada VA, Garzon A, Thode G, Daga RR, Cruzado L, Jimenez J, Sanchez M, del Rey F, Benito J, Dominguez A, Revuelta JL, Moreno S, Armstrong J, Forsburg SL, Cerutti L, Lowe T, McCombie WR, Paulsen I, Potashkin J, Shpakovski GV, Ussery D, Barrell BG, Nurse P, Cerrutti L (2002). The genome sequence of Schizosaccharomyces pombe. Nature.

[B33] Cryptococcus neoformans Sequencing Project. Broad Institute of MIT and Harvard. http://www.broad.mit.edu/annotation/genome/cryptococcus_neoformans.

[B34] Kuramae EE, Robert V, Snel B, Boekhout T (2006). Conflicting phylogenetic position of Schizosaccharomyces pombe. Genomics.

[B35] Coeytaux K, Poupon A (2005). Prediction of unfolded segments in a protein sequence based on amino acid composition. Bioinformatics.

[B36] Gregan J, Rabitsch PK, Sakem B, Csutak O, Latypov V, Lehmann E, Kohli J, Nasmyth K (2005). Novel genes required for meiotic chromosome segregation are identified by a high-throughput knockout screen in fission yeast. Curr Biol.

[B37] Decottignies A, Sanchez-Perez I, Nurse P (2003). Schizosaccharomyces pombe essential genes: a pilot study. Genome Res.

[B38] Chen D, Toone WM, Mata J, Lyne R, Burns G, Kivinen K, Brazma A, Jones N, Bahler J (2003). Global transcriptional responses of fission yeast to environmental stress. Mol Biol Cell.

[B39] Mata J, Lyne R, Burns G, Bahler J (2002). The transcriptional program of meiosis and sporulation in fission yeast. Nat Genet.

[B40] NCBI Protein. http://www.ncbi.nlm.nih.gov/entrez/query.fcgi?db=Protein&itool=toolbar.

[B41] UniProt Knowledgebase. http://www.expasy.org/tools/blast/.

[B42] Burge C, Karlin S (1997). Prediction of complete gene structures in human genomic DNA. J Mol Biol.

[B43] Milanesi L, D'Angelo D, Rogozin IB (1999). GeneBuilder: interactive in silico prediction of gene structure. Bioinformatics.

[B44] Hua S, Sun Z (2001). Support vector machine approach for protein subcellular localization prediction. Bioinformatics.

[B45] Yu CS, Chen YC, Lu CH, Hwang JK (2006). Prediction of protein subcellular localization. Proteins.

[B46] Nakai K, Horton P (1999). PSORT: a program for detecting sorting signals in proteins and predicting their subcellular localization. Trends Biochem Sci.

[B47] Chenna R, Sugawara H, Koike T, Lopez R, Gibson TJ, Higgins DG, Thompson JD (2003). Multiple sequence alignment with the Clustal series of programs. Nucleic Acids Res.

[B48] Thompson JD, Gibson TJ, Plewniak F, Jeanmougin F, Higgins DG (1997). The CLUSTAL_X windows interface: flexible strategies for multiple sequence alignment aided by quality analysis tools. Nucleic Acids Res.

[B49] Kumar S, Tamura K, Nei M (2004). MEGA3: Integrated software for Molecular Evolutionary Genetics Analysis and sequence alignment. Brief Bioinform.

[B50] Gouet P, Courcelle E, Stuart DI, Metoz F (1999). ESPript: analysis of multiple sequence alignments in PostScript. Bioinformatics.

[B51] Rhizopus oryzae Sequencing Project. Broad Institute of MIT and Harvard. http://www.broad.mit.edu/annotation/genome/rhizopus_oryzae.

[B52] Coprinus cinereus Sequencing Project. Broad Institute of MIT and Harvard. http://www.broad.mit.edu/annotation/genome/coprinus_cinereus.

[B53] JGI Phanerochaete chrysosporium v2.0. http://genome.jgi-psf.org/Phchr1/Phchr1.home.html.

