# Supplementary material for: Fungal CSL transcription factors
Source: BMC Genomics. 2007 Jul 13;8:233. doi: 10.1186/1471-2164-8-233 (PMC1973085; doi:10.1186/1471-2164-8-233)
Supplement: Additional file 1 — New and corrected fungal CSL gene prediction models [file 1471-2164-8-233-S1.doc]

**New and corrected fungal CSL gene prediction models**

###### Schizosaccharomyces japonicus

**SjCSL1**

<http://www.broad.mit.edu/annotation/genome/schizosaccharomyces_japonicus>

GenScan prediction

Supercontig 5 (bases 726033-727712, complementary strand)

ATGGGCCTTCATTCCTCTACAAGATATGATAGAGCTTCACCTGAGTCGTCTATGCAGCTGTTTTCGCTGTATGCTGCCGTCAATTCTGCACTAAGTTCCTTAGAAAGAAAAGACCGACAAACCAATGCGATACAAGCATTCCCTGAAAGCGTAAGTGAAGCTTCTACGGATTCAGAATTGCAGCGGCAGCAACAGCAGGTGCCTCAAAGCGTTCCTCCTGCAGTTCTTTTCTCACCGTCTTCAATACCGGAAAATCCAAAGAAAAGAAAAGCAAGTTTTTCAAATCACCAGGACCCTTTGATTGCACAAATTCGAGAAACAATTTTCGATCACTTAAATCTACTTTTAAAAAAGGGCAGTGCTACTTCCAATGCCCTCTGTAATCGTATACGGGATCCACTGAATCAGTACAACCAAGCCAATAAGCTGGTCACCATTTCCTATCAGCATGCCTCTGTTGCACAAAAGTCCTATGGTGCAGAAAAACGTTACCTCTGTCCGCCTCCGCTCATTTCTATAACTGGCAACACTGGATCAATATTGGGTCACTCCTTTTCAGTGCAGCTAGCCATCACAAATGAGCAGGGTCAACACTCGAATCAGGTGTCTGAAAACTTTGCTGAACGTCAATCAGTCGCTTTTCGTTCTCTTCACATTTCCTCTTCCATTGCTGCCAAGGCAAAGAGTTTTAATCTCAACATAGATGTCATAAGTTCTTCAGACGATGTTTTGGCTCAAATGGTTACAAAACCCATTAACATTATATCTAAACCATCAAAAAAGGGAAGCAAAAACCGTGTTTCCAATTCTACTCTCATGTCTGGCGCCATAATCAGTTTATACAACAGAATCAATTCTCAGACTGTTCGTACAAAATACATGTCTGTTGCCAATGGACGTTTCTGCTTGCGGAACGAATCTTGGACGCCTTTGCAAATTCGTGTGTACGGTGCCAGTGAAAGTTCATTTCATCAACCCATCATGTATGGTTCACAGGTTGTTCTCTACAACGAGCGAAGCGGTATTGTGAGCGATCCGCTGTACATTCATCGGGTTGACAAGGATCAAATTGCACCTGATGATGGTTATCTTGGGCAAATGCATCGTATCGTTCTTCGTCGAGGCCAATGCAACAACGAACCCAATGTGTTGAAACCATACGCTAATCAACAAGGCATGGATTTTGTACAACCACTTTTCCTCGGGGCAACAACAGCCCAAGAAAAAAGTACTACCGGCGACATGGACTTTCCCGTCGAACTGGAACCGGCTACTTCAATTGACGAGAGTGGTTCAATCCGGGTCAGTGATGCAGTTTGCTGGACCGTTATTGGTATTTCCCACTATGAGTTTTCGATGATGAATGCTCTTAACGCTCGTATCACTCAACCTATTACACCGTTTCCAGTAGTCGAAACATCCCCCAAGTACATTGGGGAAACACATTCACTGGAATTGCTGGTGAGTGGTTGTACCCCTAAAACACAAGTTTGGTTGGGTCCTTACGGGCCCTTGGCATATACCAGGCAGGAAACAGAAAAAGCAGACGAGAATCTGCTTATTGTCTCTCTTCCATACATTGAAGTGAATACTACAAACCCGTGGACAACTTTGCCTTTGCTCTTCACTCGGCCAAACGGACTTATTTACGTGGGCAAGTGTGACGTTGTTGTCCGCTAG

###### Schizosaccharomyces japonicus

**SjCSL2**

<http://www.broad.mit.edu/annotation/genome/schizosaccharomyces_japonicus>

GenScan prediction

Supercontig 4 (bases 1104530-1107169)

ATGTCGGGCTTAAAAAATCATTCTCCGTACGGAATTGCTCCAAATACAGTAAATTCAGAAACGCAAAATGTTGCCCGGCCGCAAGATGAAAATTCAAACTGGAACATGGAAGTCGCTTATGACCGACTGGAAACAAGAAGCCCCTTAATTGCGCGGTTGAATACTGTGTGTCCTCCTACATATATGAACCCGCAGGCACCTTGTTCTACATACGACGATTCCAATATTACTGAGCAGCAACGCTACTCCTCAGGCTTTGTCGCTAGCAGTGCCGACCCCTCACTCTATCCCTCACAAATCCCTCGCGTGGGTTACTACGTGGATAGTGCCGATCCCTCCCCTTATGCAGACGACACTGGAATTCGAACGCCTGGCAATGAAGCCAAGCTTGGGATGGCTTCAGAGTTGCCTCAAATGGGTATGAACAATGATGCTAATTCCGTGCTACGACAACAACAACAACAACAACAGTCGCATCAACAGCAGCTACAGGCACTAACAAACCAATCTGCCTTTAACCATATGGGTTGGGACGTAGCTGGTCACTTTCTGCCTGGTGACTCTGGTCGTTTTCCCGCCGTAACAGACGCATTTTCGAGTCAATTCGTTCCCTTGCAACATCCCCAACACGATACTAACCCTTACCACGGACTCCCTAATGCAGGTGCCTATCCTGAGGTTTATCCGGCCGACCCGACCTCGTCGTATTCCGGCCCATTCACTGATCAGCAAGGCAATTTCGGCTCATCGTTCGGTGACGTCGTGCCCGGCGGAAACCCAACATCTTCGAACTCACCAGCAGTGCTAGAGCTGTCAGGACACTCACCGACCCATCTCTACGGTGCTGGTGATGCTCCTCGTCTGCAACAACTTCACCAGCAACCGAGACAGCAGTTCCATGTTTCTGACTACGGCCAGTTTCACGTCCCAGCTGGAACCAACAACCCAAATGCTAATAATGGCTTTCGAGTTCCGTTTGGTGGGTCCAGCCCCGGCCCAGAGTTGAGTCAAACATCTTTCTATGACGGGCCAGAGTTTCCAGCACCCGCTGAATCTTCACATGCTTCCCTTTTGAGGCCCTCTACGTCTCACTCAGACTCCATGGGCTTAAACGCCCCCGACAGAAACAATCTTTTTCAACGGGTTAAAAATGTTCTGCGACACCCAGAGCTGTTGTGCGCTATGAAAGTATTCATGCCCTCCCTGGGTCAGAAGTCGTACGGTAAGGAGCGTCGCTATATATGCCCGCCTGCGGTCGTCTACTTGCTCGGCAGCAGCTGGTTTCGATGCCCACTCGACAAAATAAATATCATTGCGAACGCGGCTGATGACCCTGACAACCTTAAAACGTCAGAGACGCCAACCTTTTACACGAGCTCTTCCGACAGCGCCACAAACCTTCTTTCGCTGGGTCAGGTGAAACTCGACGATCCATTGGAGCAACAACAATCACCATCTCCCATATGGGCGAATACGGTGTTGAAAACGCTTTATTACTCCGGTAAGGGGGATCACAATACGTATGGCCGCTCAACTACCCTGCAGGTGCATGTTCGGACCCCACAAAAGCGGATAACTATGGATAAGCTTCGTATTGGCATCATATCGAAGCCGAGCCAAAAAAAAATGATGATGAAAGTTTCGGATTTGAATATCTGTCACGGCGATTGTGTGAGTTTATTCAATCGGTTTCGCTCTCACAACAACCTCCCACGGTATTTGTGCACAAATGTCCTTAATGACGTCGTTACAAAGCGAACAGAACACTTACAATTCCATGAGGAGTTTACGCCGGCCACAGACTTAGACTTAATGGAAGCGTCAACATGTCGATTAATTACAACAAACACCGTTTGGGAGCCATTCAATATATATTCTGTGGAAGAGCTTGAAAACAAATCAAGTTATGATCGGCGTAACACAGTTATTTGCTCCAATATGCGCATTATAATCCAAAGTCAAATTACGGGTGTGCGTTCACCTCCGCTCATTATTCGGAAGTACGAGAACCGTAAAGCACTTGTGGTGGAGGATGACCAGTTGGGCGACTCGATCAATTGTTTGTCTCGATTAGCGTTCCAGTGTCCTCGTACAAAGTTATTTTTGAATTTGGACGAATTTAGCAACGGTGAGATCCGATTTCTATCGGCTGACCCGGCTCCTGGAGAGGGTGATGGAAATGGGGAATATGTGAATCTTCCGTGGTCTGCAGTGTGGTCTATCATTACCACTCAGTCCGTTCGAACAATGTTTTTTGACGACTGTTCCGGCAATGATGGCATGCTCTCCATCCCCTCAGCACCAATCATCAAGTTCATTCGCATGGATGAGAGCAATATGTTGCATGTGTATGGCGTGAACTTCACCGCCGACGCCCAAGTCTGGATTGGTGAAAATCCATGTCAGACGTATTCGGTGACAGATGTCGAAATCGACGAGCCCACACTGCTTCGTGGACTTATTTCTACTTCACACGTACCTCCGCGACTGCATGCATATCTTGCAGATTTGGCTGATATCATTTGTCAGCCGCCGGCGCTAAGCGTAACTACATCTCCAGAGTTACCGATACTCGTTTCGCAACAAAACATCATTTTCCACAGTGGTTTCACCTGGCCAATCCATCCACATACCCTGTGA

###### Phanerochaete chrysosporium

**PcCSL2**

<http://genome.jgi-psf.org/Phchr1/Phchr1.home.html>

Manually corrected (predicted introns are shown in red lowercase)

GenScan prediction from Scaffold 6, Contig 19 (bases 50978-54385, complementary strand) with manual definition of the 4^th^ intron according to a protein sequence alignment.

ATGGCGCAAACATCTGCAACCTCCTCTGGATATTCGTCCTGGCTGCCCCTGTCGGCAAACGCCGGCGCTGAACGCCGATCAATGGgtgagtgtctatagaatgattgctgcagcacactcctcctcctcctcctcctcctccccgcccccgcccgcctcgagAGCACGGGCTGACGACGGCTGTGTCCCCCGCAGACATCCTCAAGCCTCCGTCGGCCCCCCGCAGCGCACGGCACAGGAGCATGTCCATCGACTTCACCCCCACCGGCCCCCACCAGAGCATGTTCGCCCAGCCGGACATCGACCAGCCCTTCAGCTCCCTTCAGGGGGGCGACCTCGCCCACTCTGGCTCCCACATGTCTGTCGGCGGCCACCCGCTCGACAGGAACGACCCCCTCGACATCGACACCCATGGCAGCTACGACATCTTCTCCTCCTCCAGCGGCTCGCTCGCGTCACAGAGGTACCGCACCAACGCGTCCTCCTCCTCCTCCCTCGGCCCCAACTACTCCCTCGGCGTCGATCCCATCTACCAACAGTCGTCCTTCTCTGACAACATTTCCTCCTTCCACTCGTCCAACTCGAACCCCTACGACCTCATCGGCAGCCTCTCCTCCTCCTACAGCAGCGGCAAGCCATCGCCCATCACCCCCAGCGATGTCAGCGCACTCCCGCACTCCTCGGGCTTCCCGTTCTCCAACGGCCAGTCCAAGGACTTCCCGCCGCACCACAGCTACCACGAGCCCATGCTCGACCGCCGCATCAGCAACGCGTCGGGCTACTCGAACGACTTCAACGACGAGTTCGGTTCCATGGGCGTCAACCACGGTCTCGGGCTCAACGGTTTCCCTCCCTCAGGACTTCCACCGTTCCCGGACCGCCTGGGACGCGTCCAGAATGAATCGCGCTACCCCAACTCGACTGTGCCACCCCTCACAGCCACGTCTCACCTGACACAAAGTCACAGTCCCGAGCTCATCAGAGGTGTCGCGCCGCAGGCGACCCATCTCCCGTCGTTTGACGACATGAACTTTATGGGCGCGAGCCCGACCGTCTACGACCCGCCGCTGCGTCTGCCCGCCAGCGTCAACGACGACATGGCTCGTCTGCGCCTGCAGGGTTCTGGTGACCTGCAGACCTTTATTCGgtacgtcttctctgcagttagcagtttggaaccaagcctcatgatcctctccatttcctagGCCATATCTGGATCAGTACATTAGGACGCCCAACCGTCTGGCGTTCGGCGAGCGCACGGTTATCGTCATGTCGTCCAAGGTCGCCCAGAAGTCATATGGCACAGAAAAGCGgtacgtaacaccaatttgttagcagaccgcgtgacaccgattgtgcttcctactcctcagGTTCCTCTGCCCTCCACCGACTGCTATCATGATTGGTAACTCGTGGTGGTCTGATGTCCATCGACGTGGCGAGGAGCCGAAACTTTGCCCGCCAAGAGTTGTCGTCTCCATCTCCGGGGAACCTGCACCGCAGGAGGGTTCGATCGAGTGGACGAGCGCCACGGGCAAAGCGTTCGATGTGTCTGACCCGCCAACGGGTACCACTTACATTGGGCGCTGTGTCGGCAAGCAACTCTTCATCTCCGACGTTGACGAGAAGAAGAAGAAGGTCGAGGCACTGGTCAAGATCATGGCGCCGTCTGCCGACGACGAGCCGGAACGTGTTATCGGCACGTTCCCCAGTCGTCCGATCAAGGTCATCAGCAAACCGAGCAAGAAGAGGCAAAGCGCGAAGAACCTTGAGCgtaagtgtccatgccgtactttttttgagttcacgctaataatgcggcttgctggcacagTCTGCATCAACCACGGTTCCACCATCTCGCTGTTCCACCGTCTCCGCTCCCAAACCGTCTCCACAAAGTATTTGTGTGTGTCTGGGTCGGGCTCTTCGTTCAAAGGTTCCGATGGCGCTCCTCTCATGGGCCTGGATCAGCGCGCCCGTACGCAGACACCGTCGTTTATAGCGCGGACTGCTAGCTGGGgtgagctctaacaccgctatttgacgcacagggccaacttctaacgtctgacattagATCCATTCATTATGTACATCGTCGACGTGAACAAGCCTACGGGAGGCATCGATACGCCGCCACCCCCACCGCCTCAGCCGGAGTTCCCCTCTCCTCCGCCTAACGCCATTCCCTTCACCAACAACGGATCCCAGATACCCATTTACTACAATCAGACCGTCGTCCTTCAGTGTCTGACTTCGGGCGTGGTCAGCCCTGTCCTCATCATCCGCAAGGTCGATCACCAGACCACGGTAGTCGGCGGAGGTCTTCAGGAGGGTGCCAAGGGTATCGCCGATCATTATTGTTGCCCTGGTGAGGTCTGCGGGGACCCGGTCTCGCAACTGCACAAGATCGCGTTTGAGGTCTACGACCCTAACAAGGGAGCGCCCGAGCCGGGCACCCCGGGGGTTAGCGGCGCGTTCCTGTCCTGCATGGGCGAGAAGGTCAACACCTACCGCCCCGTGGAGGGCCGCCAGTGGAACGCGAACGTTACAAATACCGCCGAGGCCGACTCCCCTATTGCTGCGCCCGGCTCGCCGATCGCTGGCACGCCGACTTCGGCGAACGGCCACGGCGACTACTTCGGTGGCAATGGCGGCTCTGGCAGCGCGCCGAACTCCCCTATCCCGACCGAGTTCCCCTCAAGCGATGGCGGCAAGGTCAAGAAGGGCAAGCGCGGCTCGTCGAGTGCCGGCGGCCTCAGCAAGCCTGCCTCGCAGAAGGGTCGCCGACGGCCAACGTCGGCGGGATCCGCCAGCGGCTCGATCTCGTCGAGCAGGCGCGGTTCGAGCAGCGACTCGTCGGCCTCCTCTGGAGCTCTCTGGCAGGTGGACATTGGCGAGACAAGCGTCTGGACGATCGTTGGTGTCGgtcagtatacttcccggctttgctgttctaatcatggctgacctcggtccgcagACCAAGTCCGATACAACTTCTACGTCCCGCCCGTGCTCTTCGACAACCAGAGCGCGCCCCAGACAGGGTCGTTCCCCATTCCCTCAAAACCGGTGACCCCCTTCCCCAACGTCGTCAAGTACCTCCCGCCCGATCGCGCTGCGGAGGCTCCCAAGCCGTGCCCGCAGTCGCGTGCGATGATGGCGAAGCCAAACCCTCACGCGTCGAAAATGCTCACCGTGTACGGCGAGAACTTCTCGAAGACGGATCCTGTCACCGTGTTCTTTGGCTCAGACCCGTCGCCGTACGTTGAGGTGCGGTGCACAGAGGTGCTCGGCTGCCTCCCACCCGAGTCGAACAACATGAAGCGCAGGCCCATCATCCTCGTTCGGCACGACGGCGTCGTGTTCCCATCCAACACCTACTACCCTTGA

###### Rhizopus oryzae

**RO3G_11583.1**

<http://www.broad.mit.edu/annotation/genome/rhizopus_oryzae/>

GenScan prediction (predicted introns are shown in red lowercase)

Corrected gene model RO3G_11583.1. Two additional 5’ exons were added to the annotated model based on the sequence alignment with other fungal CSL genes. Moreover, a possible frameshift was corrected by removing A1011 from a stretch of five adenines, which restored a conserved block of amino acids.

ATGTTTACAACTGAAACAAGAAAAAGAAAACAAGATGAAATGAATACAACTTTGCCAACCAATTGGACAGATTTTATTTATTCTACACCTTCATCACCTTCGATAGATAACTTATTTGATCAACATTCTTATTCATTTGATTCAAGCAGTGGAACAAACAGTCGTCGTCATTCTGTTGCTGTTGGTGAATTAGATTATCATTCGTTTGATTTAAATAGTTTATTAGAAGAAAGACCTTTACATAAACGTGCAATGTCATTAAGAGAAGATGATTTGACTGCCAATTTGTTTTCTTCTTATCTATTTGATTTGGTTGATACAAGACCAAGAGAGTTATCGATGGATAGCAGTATTATATCTGACCTTTCTTTGAATGATTTATCAAATAATAATCCTGACTTGTACAAGTTTAATACTTCTTTGGAAACTATTACTCCTTCTGCTACTCTGACCAACGAAATCAATTCAATGGCTGATTGGTTATTAGAAAATCCTCAAAAGAGACCAAGACGTTCTACTGATTCACCCCTTGGTTCTTCTTCTGATTCTTCTTCTTCACCACCTATTACCCCCATGCAGCAGGTCTCCTTGGGATTTGAACCTATTCAAGAAGAATGGGATCTTCAACCCTTAATTCAAAATTATTTACTTCAAAAACAATCAAGAGAAGACTATATTCCTGGTGAAAGAACAATCATGATTCTTACTAGTAAAGTAGCTCAAAAGAGCTATGGTACCGAGAAAAGgtaacacgaaaattagattagataatgactctttgcttatcattcacagATTTTTATGCCCTCCGCCTGCAACCATCATGAAAGGTACCAATTGGTGGACCTCGGACAAATTGACCGATAAGAAGACGCCTTCTTTATTTCATTCTCCGTCAAACGCTCTTCAATCACCTAAACTCACGATACATATCTCGGGTGAAACGATTCAGCAGACGGGTGTGATCGAGTGGCAGACATCTTCTGGTAACATTATCGATAACAATGCACAAAAGGTATTCGGAAGATGTATTTCAAAGCAACTTTACATTAATGACGCAGATGAAAAGAGAAAGCGTGTGGAGGTCTTGGCAAAGATCCAATTAGGCAATGGTTCCAATTTGGGTACTTTTTCAAGTAAGGGTATCAAGGTGATCAGTAAACCTTCAAAAAAGAGACAGAGTGCAAAGAATATGGAATgtgagtgacttttgtttctattgtatgatgacttatttgttttagTATGTATTCATCATGGTACAACCATCTCTTTATTCAATCGAATTCGGTCACAAACCGTATCGACTAAATATCTTGGTGTATCAACCACCACACCTCAACCCGATTCAAATGGTACTTGCTTTGTCTCTCGAACCGGTGTTTGGGATCCATTCGTCATCTGGATCGTTGATACCTCTTGTTCACCCAACACTGCCAATCGACCTAAACATAATCCACTCAATCCTAATTATCCTCCTCCACCTGCCATCGCCTTACAGACCTCTTCTACCCTAGCCATTCATTACAATCAACCCGTCGTCCTTCAGTGTGTCACCACTGGTCTGGTGAGCCCTGTGATGATCATCCGTAAAGTCGACAAACAGAGCTTGGTCTTGGGTGGTAATCGTGTGGATAATCCGATCGGATCCCTGGGTGGTGAATGCAGTGATGAAACCTTGGGTGATCCTGTCTCTCAATTACACAAGGTGGCCTTTCAAATCGTTCAGGATCCTTCATTTCATCAAGGCAACCTGAAACAAAACACAGCTGGCCACTGGAAGATTCCTCAATCAAGTCATCCAGTGACCTATTTGGCCTGTCTGAATGATGTCGTCGGCATGCACAAGACAACCTCCACCCGTCACCTTGTCCCTCAGTGTCAAGAGAACACCTTTGGCGAAATCGCACAGGATCCTATTGTGCGTCGACTGAGTACGGGTGAAATCAAACGTCGGGGCAGTTTAGGAAAGGGATCGTCTGCACTCGACCCTACTGGGCTTGAAGGTGCTTGCTGGACTGAAGATGTTTCAGATGCAGCTGTCTGGACGATTGTTGGCACGGATTGTGCTAGTTATACTTTCTGGACACCGGATGAACGTACGATGCCCACCGCACCTTTCCCTGTTCTTCATGAACTCACCAAGAAAGGCAAGGATCGCTTGACACTGACCGGCGAGAATTTGAGTCCGGATATCGAAGTTTGGTTTGGTGATGTGAAATCGACCGAGACAGAATTTGTATCTCAAGATTCGGTACACTGTAAAATTCCTTTTGATGTTGCAAATAGTACAACAATTGAACAAGAAAATGATCACAGAAGGATTCCTTTACTCTTGGTTCGTGGTAAAGGAATTGTTTACAAAACGAACCTTTATTATATTTTATGA

###### Rhizopus oryzae

**RO3G_07636.1**

<http://www.broad.mit.edu/annotation/genome/rhizopus_oryzae/>

Manually corrected (predicted introns are shown in red lowercase)

Corrected gene model RO3G_07636.1. A different 5’ splice site (GC) was chosen in order to restore an absolutely conserved part of the DNA-binding region. In addition a similar splicing pattern is found in other *R. oryzae* and *P. chrysosporium* genes.

ATGAATTATCCAGAATTATTAACAAATGAAGTTGAATCTATCCCATCCTCTTGGTCTTCTAATTCATCTTTACCACATGTTTCTTTATTCTCTCCAAGTTTTTTAGAAACATTAAAATTAGAAGATGAAAATGATTTTAATACAATACATCCTTCTGTTATCTATCATCATTCACCTCAAACCACCACTATTTCTTCACCCGATCATTCCAATCTATTTAATTTACAAGAAAATAAAATTTATTCAAATCATCAACAAAAGAATCTCATTCAACATTATCTATCCACCAAACAAGGAGAAAAGAAACTGACGATTTTGACGAGTAAAGTAGCTCAAAAGAGTTATGGAAATGAAAAAAGgcaagtggtgaaataactcacttctcattaaaactcatctttcccatcagATTCTTGTGTCCTCCTCCTTCTACTATCCTTTCTGGTACGGGTCACTGGTGGACAGCAAAACAACATCCACCGAATCTGACAATTCAAATCTCTGGTGAAAAATTAAGTCATCAAGGCACGATCGATTGGTACAAGGATGGCAATCTGTTGGATCAACCTTCTGCCGTTCTTTTAGCAAATCAAGGAAGTAATCTGATTGGTAATTGTGTATCCAAGCAATTGCATGTGAGTGGCGCAGATGAAAAGCGAAGGAAAGCGCAAGTTCAAGTCGAGATTCGATCAGGTCAAGGTACACCGATCGGTATCTTTCATTCAAAACCTATCAAAGTGATCAGTAAACCATCCAAGAAGAGACAGAGTGTTAAAAATATGGATCTCTGTATTCATCATGGCACGACTGTCGCTTTATTCAATCGTGTACGAGCTCAGACCATCTCCACAAAATATCTCGGTGTTTCTTCTCTTGACAGTCAACAAAAGGACAGAGGTACTTGTTTTGTCACTCGAACCACCTCTTGGGATCCATTCCTCATCTACATCGTCGATCTCTCTCGTTCTCCAAACACACCTTCTCCAGTGCCATTCAGTCATCATCCCACCATCGATCACTATCCTCCTCCTCCTGCTATCGCCATTCAAAACCATCAGGGCTCACTTGCCCTTCATTATAATCAACCTGTCGTATTACAGTGTGTCTCTACCGGCCTGGTCAGTCCGGTGTTGATCATCCGTCGAGTAGAGAAAGGTAGCATGGTGATGGGTGGTAATCGTGTGAATGATCTTTCTTATCCTACAGGAGGTGAGTGGGGTGATGAAGCCCTGGGTGATCCTGTCTCTCAACTTCACAAAGTCGGATTTCAAATCGTTCAAGATCCATCCATCGCCCAATACAACAAGTCTACTTTTCAGGAACAGGACAAGTTCTTTTTACCCCCTGTCACCCACTGGACCTTGCCTCAGGCCACTTCTGCTATTAATTATCTCGCCTGTATTCATGATGTCGTCGGAATGCATCGAGTGACGGATGAGAGAAAGATCGTGAGTCGATTTACAACAGAAATCGAAGACATCAAGATGGCTGTTCGTAAACGTCGTCTGTCCTATCAGCAACACAGTACTACAGTCAAAAGCTCTAATCGCCGTCGAGTGAATTCTTTAAATGATGAATTGCTATCAAGACATGTTGGTGGCGACGCTGGTCGTTGTCCTGATCAACCCTTAAATGGTGATTGTTGGACTGAAGATGTTTCGGATTCAGCCGTCTGGACGATTGTCGGTACCAATTCGACTAGCTTTGCCTTCTGGACACCTCCTGATTATTCTAAACCATTCTTTGATCTTCAAGATTTTCCCTATGTTGACTCTATCCAATCATTATCCAGTACAGTCTTGTCTCTCGTAGGTGAACACTTTACATCAGACCTCACCGTTTGGTTTGGTGATGTGCCTTCCATCCAGACGGAATTCAAATCAAGTCAACTTCTCTCCTGTACTGTGCCTGAACGACATGAATTATTGGATAGTTTTGCAACTCAGTTAGACCCCGACACAAGCAGGCATAAAATACCTCTTCTTTTGGTTCAAGAAGATGGCATCATTTATAATTCACTACTGTTTTATTCATTTTAA

###### Rhizopus oryzae

**RO3G_14587.1**

<http://www.broad.mit.edu/annotation/genome/rhizopus_oryzae/>

Manually corrected (predicted introns are shown in red lowercase)

Corrected gene model RO3G_14587.1. A different 3’ splice site was chosen for the 2^nd^ intron in order to restore a highly conserved part of the DNA-binding region. In addition a similar splicing pattern is found in other *R. oryzae* and *P. chrysosporium* genes.

ATGACTGGTATCCCTCCTCAACATGATATCATAAAGCACAGTATCATATTTCACAATAATGACACTAAGCCTCAACCTCCTTCTCCTGAAACACCCACAAGCAGCAGCAGCAGTCGAAAACGTAAACAAGATTTTCAATTCACTCCTGATCACCTTTCCTTTCCTCAATACAATTATGCCGAACCTGAAACACCTCTGGCCTTACAACACGAAAAATTCATTCAATCTTTACATCCAGATGGAAGCGTGGGAGAAAATGAAGAACTGATGGTCGTCAACTTTGATCAATCCAATCCATTCCCAAGGCTAACTCAACCGATTGACCTGGATGACCTCCTTCAGCAAAGACAAGCCTTTCAAACCTGGGACGCTTCTTCTTCATCTCCTATTCAATCTCCTACTCGTTATTCCCCAGGAACACCAGGTTTTTTCACGCCAGGATTTTTAGAGTCGCTTCAAGAACATCCAGTCTATGACCATTCGCTTTCGATCGATTATGGTTCACATCATTTCAATCAGGAATACAATCCACTTTTAGTCAAGCTGGAAGAACAGgtatttttaaaattcaataaacaaaaacactcatttcttaccagTCACCCGAAAAGAATTTGGTATCACAAAGTGGAGAAAGTGTCACGTCTTTATTCCCTTCTGATCCTGCTTCCATAGTAAGACCCAATCAAGCGCACGACAGCCCCATCCGTCGATCCTCCAGTCACACGACGGCCAGCAGCCCACACCGTCTTGTGCATCTCTCGCCACTCAAGATCAAACCCTTCATTCAGACTTACCTTGCACACGCCATCACCCAACCCGCCGCGACTCAGCTGGGTGAGAAGACGGTGATCGTGTTGACGAGTAAAGTGGCCCAAAAGAGTTATGGCACAGAGAAAAGgtaagcgatcggtgacaagagccccaaaggactgacagcccacagGTTCCTCTGTCCTCCCCCGACAGCCATCTTGGTCGGCACGAGCTGGTGGACGACGAAAGAAAAGATCCAAGACAAGGAGGAAACGTTACGCATCCCCAGCCTGGAGAAGGACATTCTCTTGGCGCCTCCTAAACTGACCGTGTCCATATCAGGTGAGACGTCGACGCAAGCGGGACAGTTGGAATGGTACACGGTCTCTGGTGCCACCGTCGGACAGACCGGACAGATCAAGCCTCCGATCAAGCCAGAATCCACCAGCCGTTTCCGCAGTTCAGAGTCTCGTCATCCTCCGGCGGACGCCTACAGCAACGAACGTCAGGAACTCTTGGCCGCCGGAAAATCCGTCTCGAAGCATCTCTATATCCATGACGCGGACGAGAAGCGAAAACGCGTCGAGTGCCTGGTGAAGCTTCAGTTGGCGAATGGACTTCAGCTCGGTCCACTGGCCAGTAAGGCGATCAAGGTGATCAGTAAACCGAGCAAGAAGCGACAGAGTATAAAGAACATGGAACgtaagcatcctctctctctcttttctaccttccttactgaccctttttcttctcagTCTGTATCCATCATGGCACGACCGTGTCCTTATTCAACCGGATCCGTTCCCAGACCGTGTCGACTAAATACCTCGGTGTCTCGACCAGCAAAGGCAGCCCGCTGGCTTTCCCCGGCCTGGCCTTTCAACACGAAAAGAACCGGACGAGCGAAGGGACCTGTTTTGTGGCGCGAACGACCAGCTGGGACCCGTTTGTGATCTGGATCGTCGATACGTCAGCCAGCAGCGAAGAAGAAGGCGAGACGCCAGAGGATTATATCGGCCATCATGTGTTTGCCCGAAGCACGCCCTACCCACCCCCGCCTCCGATCGCGCTCAAGAACAAGACGGGCGGGCCGGTGCCGATTCACTATAACCAACATGTCGTCCTCCAATGCCTGACCACGGGCCTGGTCAGTCCGGTGATGATCATCCGCAAGGTGGATCGTGCCTCGACCGTGGTCGGCGGCGCCCGAGACGATGTGAGTGGCAGCGGCGGCGAATTTGGCGACGAGGTCTTGGGCGACCCGGTGAGTCAACTGCACAAGATTGCGCTTCAGATCGTGCAAGACCCCAAGATGAGTGTAATGCAAGCCCCCGACCCTCGGATGCCGCGCACCTCTCAGCCGGTGACCTACCTGGCCTGCTTGAATGACATGGTCGGCATGCACAAGACGTCCGAAGGGCGCTCGTGGGCCGGCTGGGACGACAGCATCACCTCGCAGGAAGGAGGCAAGATCATCCGGAAACGTCGGGTGTCGACCGACGTCCAGCCCGAGACCCTGATGTCTTGCATGTCCCTTTCAGATTACCCCCGACGTCGGGTCAACAGCCTCGAGGACCCCGCGCCTTATCTCGCCCGCAAGTCGAGCGTCAGCAGCCTTTCTTCCACCACCAGTCGTCCCCACCTCGGCGCCTTTTGGAGTGAAGACGTCTCGGATGCTGCCGTCTGGACCCTCGTCGGCACCGACTGTGCCACCTACACCTTCTGGTCGCCCTTCCTCGACGATCCCTCCACTCCCCTCTCCACCGGCCCTTTCCCGGCCCTCTCTCACTTTTTTACATCGACCAACAAGTTGGATCACGAACGGTTCCTGACCATGCACGGTGAGAATTTCTCGCGTGATCTCCAGGTCTGGTTTGGCGACGTCAAGGCGAATCACACCGAATACCGAAGTAGGGAATTGATCATCTGTAAGGTGCCTCCGAGACATGAACTGATGGAGGTCAAGAAGGTCTATGGTGATCTTCCGATTCTGTTGGTGAGAGGTGATGGCACGATATGTAAAACCGGTAAATGCTTTTCGTTGTAA

###### Coprinus cinereus

**CC1G_01706.1**

[http://www.broad.mit.edu/annotation/genome/coprinus_cinereus/](http://www.broad.mit.edu/annotation/genome/rhizopus_oryzae/)

Manually corrected (predicted introns are shown in red lowercase)

Corrected gene model CC1G_01706.1. A predicted intron (underlined) was included in the coding region in order to restore a highly conserved block of amino acids. The second intron seems to be rather long but is supported by several software predictions and unfortunately occurs in a poorly conserved region, which hampers making decisions based on a protein sequence alignment.

ATGAAGCCATTGACTTCGACATTGGCTACGGGACCCGCTTCCCCCATGAATGTGGATCAGGAGGACCCTTTCAACCTTCTCACATTCTTCCCCCCTGGGATTGGACGGCGAGACGACTGGCACTGGCTGAGAGGGGAAGCTTTAGCAGAAGAAGTCGAAGAAGCGTCAATAGATGTCGAAGGTGACGATGGAGGAGACTCTCCGGCAATATTCGTTCCAACGGAAGACAGGCACATGAGCGAGGCGATAAAGGGGGAAGATAAATTAGGCATTTTGTCGTTCGgtatgtgacagttgtgtaatgggaacgtatctgactgtggtcgcagGGTATCGCGACAGTCACAACGATGACGACGATGAGGACGACCTGGACGACGTCGAAATTGTAGACGAGACGGGCAGGTTCGAACCTGAGATTGAGACGTGGGAAGACTTGTATGAAGCGCACCGGCGTCGGCGGATGGGTGGGAAGTCGgtgccggcagtggagccatgttcggagctattcttcggagacggccgagttttgtgatttatgtatttgatggtgtttgatcacgtggagggtccgtgtgatctttgactacatttggacttcggcagtgtttggtttcccacttctctttcttttcccaccagtgtgaacggcctgtgcaaatggctgaagcaactgctcaatctttctttctgagcgaccctcatcatctccaaatgcctccagccgtcgccgtcgtcgacggcgaccccaactctcgctcggcgaccccaaacgtggcccaatctgtaaaatacctcttctctatatcctaatatcttgtaatgaattcgcctttcgacacgcttctttaggtcacaaataaacgttccttgcagCCAGATATAAATCCGAAACGTTCACCGACACCTCAAGTTGGCGGCGCACCGTCACCAGTCGTCGCTCAGGACCCCAACCTCTCAATTCAGTCCATTCTAGCCGCCTCGAAGAAGGATGGGCCTACAAATGGCACCTCCCAGGCAACATGGCACCCCCCTCCGCCTCCCGCAACCTCTgtgagcaacattctgatctttatgtcgcgtctgcctttctaacctctttcatagACCTCCACCGGGAAGCGGAAATTGGATGAAACCGAGTCAGAAGACACGCATAGCAAGATACGTCGAATTGTTCGCGACCATGTCTCTCAAGACCCCGCTCGCGTCGTGCCTATGACCACCGTAATATGTCTCCACGCGGCCGTCGCACAGAAATCCTATGGAAGCGAGAAGCGATTCCTCTGTCCACCGCCGATTGTCCACATAGAGGGCCCTGTCTGGCATCTACGCACCCAGAGCCTCTCCATGGCGGTCGTCTCTGAAACCGGAGAGAGGTCGTTTGAGCAGAAGGCGACGCTCGATAACAACATGGTCTCGAGCTTCAAGTTCCTTCATGTTACAGGAACGGCGAAAGCTAAAAGCTTCCAGCTGTCCCTCGATATCGCAGAACCCCCACCCCCTTCCCTCAACCCAGAAGGTTCCGAAACCTCGGCGAATGGACGCGTTTGGGCTACCTTCGACTCCGCCCCTGTGACCATTATCTCCAAGCCATCCAAGAAGACCGCGAAAACGCGCAATATCTCTTCTTGCATCCTGGCCGGAGGCCCAGTGTCGCTCTTCAATCGCATCAACTCACAAACCGTCCGTACCAAGTATATGACGATCGACCACGGACAACTGTGCGCAAGCAACATTGGCTGGTCTGCGTTTAATGTCAATGTCGTTCGCAGACCCGACGGGTCACCAAACACGAgtatgtatcaggtgacctatttatgaccacgtttctgaccgttttgtagACGGGCCCCAGCCAGTAACCTACGGCTGCGAAATCGTCCTGTCAGACACGCAATCCGGTATCTCAACCTCCCCTTTGGTCATCCGGAAAGTTGACAAGGGTCGCGTTTCCCCCGACGACGGAGGTCCAGTTAGCCAGATGCAGAAGATTGCTCTTCAGAGAGTGAACCCAGATGGCTCTCGGCATTACCTCTCAGCCGCAGGTCCTCTTCCGGGTACACCTGGCGTAGTGGCGCCGCCAGCTCCAGGCATGTCAACGCAGGCTGGCACACATCCTCTTCTCTTCCAGAACCCGAGGATTAGGGACGAAGTCAGGGATGGGATACGGGTTATCTCTGACGAAGTTGATGATTATTTGTGCTGGACCATTGTTGGCATTTgtgagtgtgtgttactcagtactcgagacgtattctgacctgaggctgcagCTAAATTCCAGTACACTTTCTTCGATGCTTTCGGACAAAACAACAAGATACCCGAAACACCTATCACGCCTTTCCCTACTTTGTTTACCGCCCCCGTCTACCGAGCCGCAAACAACACCATTGAACTCACCGTCTCCAATTTCTTCTATGCCCACCCTAAAACCAGGATGCAAACTCCGTTGGATGTTTACCTAGGCAGCCTGGGTCCCTTGCACACGCGTGTGTATCAGACAAGTCCCCCTGGCCCGCTAACGTCCATCTCGCCCTTCGTTCCTGTCACTCCGGTGGAGGTTCCGCCTCCTGCGGGTGATCCTAATGCCCCTGCCAATCGATATGTGTCCACTGGCCCCTTACACACCATCGTCATTGTTGAATTGCCTCCGCTGGCCGATGTCATCAAAGCCATGGAAGACGAACCAGTGCCGACAGCCTCTGACGTGTCGGGTAGCAAGCCTCACCAGGAAAGCGAAGGTCAGGAAGGCGTCCCACCGCCACCACCGCCGCCACCTTCAATGGCTGGACGAAGCCTCCCATTGCTCTTTATCCGCTCAAGTGACGGTGTAGGGTATCACTCGGGAAGAACTATAGCTTGCGAGCCGATCCATTACTCGCTGGATCTTGCAGCTATGGCTCCCAATGGGCCCAACGGTGTTGATCCGCAATGGCTGGCAGCTGCTCAGGCTGCGGCAGCCGCGGATGGAAACATGCAGCCTTGGACCCTGAGAGTAATGTAA
