# Supplementary material for: Fungal CSL transcription factors
Source: BMC Genomics. 2007 Jul 13;8:233. doi: 10.1186/1471-2164-8-233 (PMC1973085; doi:10.1186/1471-2164-8-233)
Supplement: Additional file 2 — New and corrected fungal CSL protein prediction models [file 1471-2164-8-233-S2.doc]

**New and corrected fungal CSL protein prediction models**

###### Schizosaccharomyces japonicus

**SjCSL1**

<http://www.broad.mit.edu/annotation/genome/schizosaccharomyces_japonicus>

MGLHSSTRYDRASPESSMQLFSLYAAVNSALSSLERKDRQTNAIQAFPESVSEASTDSELQRQQQQVPQSVPPAVLFSPSSIPENPKKRKASFSNHQDPLIAQIRETIFDHLNLLLKKGSATSNALCNRIRDPLNQYNQANKLVTISYQHASVAQKSYGAEKRYLCPPPLISITGNTGSILGHSFSVQLAITNEQGQHSNQVSENFAERQSVAFRSLHISSSIAAKAKSFNLNIDVISSSDDVLAQMVTKPINIISKPSKKGSKNRVSNSTLMSGAIISLYNRINSQTVRTKYMSVANGRFCLRNESWTPLQIRVYGASESSFHQPIMYGSQVVLYNERSGIVSDPLYIHRVDKDQIAPDDGYLGQMHRIVLRRGQCNNEPNVLKPYANQQGMDFVQPLFLGATTAQEKSTTGDMDFPVELEPATSIDESGSIRVSDAVCWTVIGISHYEFSMMNALNARITQPITPFPVVETSPKYIGETHSLELLVSGCTPKTQVWLGPYGPLAYTRQETEKADENLLIVSLPYIEVNTTNPWTTLPLLFTRPNGLIYVGKCDVVVR*

###### Schizosaccharomyces japonicus

**SjCSL2**

<http://www.broad.mit.edu/annotation/genome/schizosaccharomyces_japonicus>

MSGLKNHSPYGIAPNTVNSETQNVARPQDENSNWNMEVAYDRLETRSPLIARLNTVCPPTYMNPQAPCSTYDDSNITEQQRYSSGFVASSADPSLYPSQIPRVGYYVDSADPSPYADDTGIRTPGNEAKLGMASELPQMGMNNDANSVLRQQQQQQQSHQQQLQALTNQSAFNHMGWDVAGHFLPGDSGRFPAVTDAFSSQFVPLQHPQHDTNPYHGLPNAGAYPEVYPADPTSSYSGPFTDQQGNFGSSFGDVVPGGNPTSSNSPAVLELSGHSPTHLYGAGDAPRLQQLHQQPRQQFHVSDYGQFHVPAGTNNPNANNGFRVPFGGSSPGPELSQTSFYDGPEFPAPAESSHASLLRPSTSHSDSMGLNAPDRNNLFQRVKNVLRHPELLCAMKVFMPSLGQKSYGKERRYICPPAVVYLLGSSWFRCPLDKINIIANAADDPDNLKTSETPTFYTSSSDSATNLLSLGQVKLDDPLEQQQSPSPIWANTVLKTLYYSGKGDHNTYGRSTTLQVHVRTPQKRITMDKLRIGIISKPSQKKMMMKVSDLNICHGDCVSLFNRFRSHNNLPRYLCTNVLNDVVTKRTEHLQFHEEFTPATDLDLMEASTCRLITTNTVWEPFNIYSVEELENKSSYDRRNTVICSNMRIIIQSQITGVRSPPLIIRKYENRKALVVEDDQLGDSINCLSRLAFQCPRTKLFLNLDEFSNGEIRFLSADPAPGEGDGNGEYVNLPWSAVWSIITTQSVRTMFFDDCSGNDGMLSIPSAPIIKFIRMDESNMLHVYGVNFTADAQVWIGENPCQTYSVTDVEIDEPTLLRGLISTSHVPPRLHAYLADLADIICQPPALSVTTSPELPILVSQQNIIFHSGFTWPIHPHTL*

###### Phanerochaete chrysosporium

**PcCSL2**

<http://genome.jgi-psf.org/Phchr1/Phchr1.home.html>

MAQTSATSSGYSSWLPLSANAGAERRSMEHGLTTAVSPADILKPPSAPRSARHRSMSIDFTPTGPHQSMFAQPDIDQPFSSLQGGDLAHSGSHMSVGGHPLDRNDPLDIDTHGSYDIFSSSSGSLASQRYRTNASSSSSLGPNYSLGVDPIYQQSSFSDNISSFHSSNSNPYDLIGSLSSSYSSGKPSPITPSDVSALPHSSGFPFSNGQSKDFPPHHSYHEPMLDRRISNASGYSNDFNDEFGSMGVNHGLGLNGFPPSGLPPFPDRLGRVQNESRYPNSTVPPLTATSHLTQSHSPELIRGVAPQATHLPSFDDMNFMGASPTVYDPPLRLPASVNDDMARLRLQGSGDLQTFIRPYLDQYIRTPNRLAFGERTVIVMSSKVAQKSYGTEKRFLCPPPTAIMIGNSWWSDVHRRGEEPKLCPPRVVVSISGEPAPQEGSIEWTSATGKAFDVSDPPTGTTYIGRCVGKQLFISDVDEKKKKVEALVKIMAPSADDEPERVIGTFPSRPIKVISKPSKKRQSAKNLELCINHGSTISLFHRLRSQTVSTKYLCVSGSGSSFKGSDGAPLMGLDQRARTQTPSFIARTASWDPFIMYIVDVNKPTGGIDTPPPPPPQPEFPSPPPNAIPFTNNGSQIPIYYNQTVVLQCLTSGVVSPVLIIRKVDHQTTVVGGGLQEGAKGIADHYCCPGEVCGDPVSQLHKIAFEVYDPNKGAPEPGTPGVSGAFLSCMGEKVNTYRPVEGRQWNANVTNTAEADSPIAAPGSPIAGTPTSANGHGDYFGGNGGSGSAPNSPIPTEFPSSDGGKVKKGKRGSSSAGGLSKPASQKGRRRPTSAGSASGSISSSRRGSSSDSSASSGALWQVDIGETSVWTIVGVDQVRYNFYVPPVLFDNQSAPQTGSFPIPSKPVTPFPNVVKYLPPDRAAEAPKPCPQSRAMMAKPNPHASKMLTVYGENFSKTDPVTVFFGSDPSPYVEVRCTEVLGCLPPESNNMKRRPIILVRHDGVVFPSNTYYP*

###### Rhizopus oryzae

**RO3G_11583**

<http://www.broad.mit.edu/annotation/genome/rhizopus_oryzae/>

MFTTETRKRKQDEMNTTLPTNWTDFIYSTPSSPSIDNLFDQHSYSFDSSSGTNSRRHSVAVGELDYHSFDLNSLLEERPLHKRAMSLREDDLTANLFSSYLFDLVDTRPRELSMDSSIISDLSLNDLSNNNPDLYKFNTSLETITPSATLTNEINSMADWLLENPQKRPRRSTDSPLGSSSDSSSSPPITPMQQVSLGFEPIQEEWDLQPLIQNYLLQKQSREDYIPGERTIMILTSKVAQKSYGTEKRFLCPPPATIMKGTNWWTSDKLTDKKTPSLFHSPSNALQSPKLTIHISGETIQQTGVIEWQTSSGNIIDNNAQKVFGRCISKQLYINDADEKRKRVEVLAKIQLGNGSNLGTFSSKGIKVISKPSKKRQSAKNMELCIHHGTTISLFNRIRSQTVSTKYLGVSTTTPQPDSNGTCFVSRTGVWDPFVIWIVDTSCSPNTANRPKHNPLNPNYPPPPAIALQTSSTLAIHYNQPVVLQCVTTGLVSPVMIIRKVDKQSLVLGGNRVDNPIGSLGGECSDETLGDPVSQLHKVAFQIVQDPSFHQGNLKQNTAGHWKIPQSSHPVTYLACLNDVVGMHKTTSTRHLVPQCQENTFGEIAQDPIVRRLSTGEIKRRGSLGKGSSALDPTGLEGACWTEDVSDAAVWTIVGTDCASYTFWTPDERTMPTAPFPVLHELTKKGKDRLTLTGENLSPDIEVWFGDVKSTETEFVSQDSVHCKIPFDVANSTTIEQENDHRRIPLLLVRGKGIVYKTNLYYIL*

###### Rhizopus oryzae

**RO3G_07636**

<http://www.broad.mit.edu/annotation/genome/rhizopus_oryzae/>

MNYPELLTNEVESIPSSWSSNSSLPHVSLFSPSFLETLKLEDENDFNTIHPSVIYHHSPQTTTISSPDHSNLFNLQENKIYSNHQQKNLIQHYLSTKQGEKKLTILTSKVAQKSYGNEKRFLCPPPSTILSGTGHWWTAKQHPPNLTIQISGEKLSHQGTIDWYKDGNLLDQPSAVLLANQGSNLIGNCVSKQLHVSGADEKRRKAQVQVEIRSGQGTPIGIFHSKPIKVISKPSKKRQSVKNMDLCIHHGTTVALFNRVRAQTISTKYLGVSSLDSQQKDRGTCFVTRTTSWDPFLIYIVDLSRSPNTPSPVPFSHHPTIDHYPPPPAIAIQNHQGSLALHYNQPVVLQCVSTGLVSPVLIIRRVEKGSMVMGGNRVNDLSYPTGGEWGDEALGDPVSQLHKVGFQIVQDPSIAQYNKSTFQEQDKFFLPPVTHWTLPQATSAINYLACIHDVVGMHRVTDERKIVSRFTTEIEDIKMAVRKRRLSYQQHSTTVKSSNRRRVNSLNDELLSRHVGGDAGRCPDQPLNGDCWTEDVSDSAVWTIVGTNSTSFAFWTPPDYSKPFFDLQDFPYVDSIQSLSSTVLSLVGEHFTSDLTVWFGDVPSIQTEFKSSQLLSCTVPERHELLDSFATQLDPDTSRHKIPLLLVQEDGIIYNSLLFYSF*

###### Rhizopus oryzae

**RO3G_14587**

<http://www.broad.mit.edu/annotation/genome/rhizopus_oryzae/>

MTGIPPQHDIIKHSIIFHNNDTKPQPPSPETPTSSSSSRKRKQDFQFTPDHLSFPQYNYAEPETPLALQHEKFIQSLHPDGSVGENEELMVVNFDQSNPFPRLTQPIDLDDLLQQRQAFQTWDASSSSPIQSPTRYSPGTPGFFTPGFLESLQEHPVYDHSLSIDYGSHHFNQEYNPLLVKLEEQSPEKNLVSQSGESVTSLFPSDPASIVRPNQAHDSPIRRSSSHTTASSPHRLVHLSPLKIKPFIQTYLAHAITQPAATQLGEKTVIVLTSKVAQKSYGTEKRFLCPPPTAILVGTSWWTTKEKIQDKEETLRIPSLEKDILLAPPKLTVSISGETSTQAGQLEWYTVSGATVGQTGQIKPPIKPESTSRFRSSESRHPPADAYSNERQELLAAGKSVSKHLYIHDADEKRKRVECLVKLQLANGLQLGPLASKAIKVISKPSKKRQSIKNMELCIHHGTTVSLFNRIRSQTVSTKYLGVSTSKGSPLAFPGLAFQHEKNRTSEGTCFVARTTSWDPFVIWIVDTSASSEEEGETPEDYIGHHVFARSTPYPPPPPIALKNKTGGPVPIHYNQHVVLQCLTTGLVSPVMIIRKVDRASTVVGGARDDVSGSGGEFGDEVLGDPVSQLHKIALQIVQDPKMSVMQAPDPRMPRTSQPVTYLACLNDMVGMHKTSEGRSWAGWDDSITSQEGGKIIRKRRVSTDVQPETLMSCMSLSDYPRRRVNSLEDPAPYLARKSSVSSLSSTTSRPHLGAFWSEDVSDAAVWTLVGTDCATYTFWSPFLDDPSTPLSTGPFPALSHFFTSTNKLDHERFLTMHGENFSRDLQVWFGDVKANHTEYRSRELIICKVPPRHELMEVKKVYGDLPILLVRGDGTICKTGKCFSL*

###### Coprinus cinereus

**CC1G_01706**

<http://www.broad.mit.edu/annotation/genome/coprinus_cinereus/>

MKPLTSTLATGPASPMNVDQEDPFNLLTFFPPGIGRRDDWHWLRGEALAEEVEEASIDVEGDDGGDSPAIFVPTEDRHMSEAIKGEDKLGILSFGYRDSHNDDDDEDDLDDVEIVDETGRFEPEIETWEDLYEAHRRRRMGGKSPDINPKRSPTPQVGGAPSPVVAQDPNLSIQSILAASKKDGPTNGTSQATWHPPPPPATSTSTGKRKLDETESEDTHSKIRRIVRDHVSQDPARVVPMTTVICLHAAVAQKSYGSEKRFLCPPPIVHIEGPVWHLRTQSLSMAVVSETGERSFEQKATLDNNMVSSFKFLHVTGTAKAKSFQLSLDIAEPPPPSLNPEGSETSANGRVWATFDSAPVTIISKPSKKTAKTRNISSCILAGGPVSLFNRINSQTVRTKYMTIDHGQLCASNIGWSAFNVNVVRRPDGSPNTNGPQPVTYGCEIVLSDTQSGISTSPLVIRKVDKGRVSPDDGGPVSQMQKIALQRVNPDGSRHYLSAAGPLPGTPGVVAPPAPGMSTQAGTHPLLFQNPRIRDEVRDGIRVISDEVDDYLCWTIVGISKFQYTFFDAFGQNNKIPETPITPFPTLFTAPVYRAANNTIELTVSNFFYAHPKTRMQTPLDVYLGSLGPLHTRVYQTSPPGPLTSISPFVPVTPVEVPPPAGDPNAPANRYVSTGPLHTIVIVELPPLADVIKAMEDEPVPTASDVSGSKPHQESEGQEGVPPPPPPPPSMAGRSLPLLFIRSSDGVGYHSGRTIACEPIHYSLDLAAMAPNGPNGVDPQWLAAAQAAAAADGNMQPWTLRVM*
